# Specific chromatin landscapes and transcription factors couple breast cancer subtype with metastatic relapse to lung or brain

**DOI:** 10.1186/s12920-020-0695-0

**Published:** 2020-03-06

**Authors:** Wesley L. Cai, Celeste B. Greer, Jocelyn F. Chen, Anna Arnal-Estapé, Jian Cao, Qin Yan, Don X. Nguyen

**Affiliations:** 1 Department of Pathology, Yale School of Medicine, 333 Cedar St, New Haven, CT 06510 USA; 2 Present address: Department of Pharmacology, Vanderbilt University School of Medicine, 2209 Garland Ave, Nashville, TN 37240-0002 USA; 3 Yale Cancer Center, Yale School of Medicine, 333 Cedar St, New Haven, CT 06510 USA; 4 Present address: Rutgers Cancer Institute of New Jersey, Rutgers, 195 Little Albany St, New Brunswick, NJ 08903-2681 USA; 5 Yale Stem Cell Center, Yale School of Medicine, 333 Cedar St, New Haven, CT 06510 USA; 6 Department of Pathology, Yale School of Medicine, P.O. Box 208023, New Haven, CT 06520-8023 USA; 7 Department of Medicine (Medical Oncology), Yale School of Medicine, 333 Cedar St, New Haven, CT 06510 USA

**Keywords:** ATAC-seq, ChIP-seq, Breast cancer, Metastasis, Transcription factors, Epigenomics, mRNA expression

## Abstract

**Background:**

Few somatic mutations have been linked to breast cancer metastasis, whereas transcriptomic differences among primary tumors correlate with incidence of metastasis, especially to the lungs and brain. However, the epigenomic alterations and transcription factors (TFs) which underlie these alterations remain unclear.

**Methods:**

To identify these, we performed RNA-seq, Chromatin Immunoprecipitation and sequencing (ChIP-seq) and Assay for Transposase-Accessible Chromatin using sequencing (ATAC-seq) of the MDA-MB-231 cell line and its brain (BrM2) and lung (LM2) metastatic sub-populations. We incorporated ATAC-seq data from TCGA to assess metastatic open chromatin signatures, and gene expression data from human metastatic datasets to nominate transcription factor biomarkers.

**Results:**

Our integrated epigenomic analyses found that lung and brain metastatic cells exhibit both shared and distinctive signatures of active chromatin. Notably, metastatic sub-populations exhibit increased activation of both promoters and enhancers. We also integrated these data with chromosome conformation capture coupled with ChIP-seq (HiChIP) derived enhancer-promoter interactions to predict enhancer-controlled pathway alterations. We found that enhancer changes are associated with endothelial cell migration in LM2, and negative regulation of epithelial cell proliferation in BrM2. Promoter changes are associated with vasculature development in LM2 and homophilic cell adhesion in BrM2. Using ATAC-seq, we identified a metastasis open-chromatin signature that is elevated in basal-like and HER2-enriched breast cancer subtypes and associates with worse prognosis in human samples. We further uncovered TFs associated with the open chromatin landscapes of metastatic cells and whose expression correlates with risk for metastasis. While some of these TFs are associated with primary breast tumor subtypes, others more specifically correlate with lung or brain metastasis.

**Conclusions:**

We identify distinctive epigenomic properties of breast cancer cells that metastasize to the lung and brain. We also demonstrate that signatures of active chromatin sites are partially linked to human breast cancer subtypes with poor prognosis, and that specific TFs can independently distinguish lung and brain relapse.

## Background

Worldwide, breast cancer is the leading cause of cancer related deaths in women [1, 2]. Breast cancers are highly heterogenous and are classified based on immunohistochemistry markers (e.g. estrogen receptor (ER), progesterone (PR) and HER2 status). Resected primary breast tumors can also be independently stratified into intrinsic molecular subgroups (e.g. PAM50 subtypes) based on transcriptomic profiling. The most prominent molecular subtypes identified to date include the normal-like, luminal A, luminal B, HER2 enriched (referred as HER2), basal-like, and claudin-low tumors [3–5]. While their predictive and prognostic significance has been examined in clinical settings [6–9], the cellular and molecular determinants of these subtypes remains a subject of significant investigation [10–12].

Advanced breast cancer is associated with a high mortality rate due to the formation of metastases in vital distant organs [13]. Patients with relapses in the lungs and brain in particular, have limited clinical options and poor outcomes [14, 15]. In studies comparing human primary and metastatic breast tumor samples, metastasis-specific driver mutations are rare and mutations associated with tissue-specific (a.k.a. organotropic) metastasis have not been identified [16]. Conversely, differences in gene expression have been found to mediate metastasis in particular organ sites [17]. Importantly, many genes associated with metastasis are already differentially expressed in primary tumors. This is most notably observed in tumors resected from patients that are at risk for lung or brain relapse [13]. These unique metastatic propensities may be influenced, in part, by primary breast cancer subtype. For instance, basal-like tumors, which often share features of triple negative cancers (negative for ER, PR, and HER2), are more aggressive [18]. Breast cancer patients with prior lung metastasis are also more likely to develop brain metastases [19, 20]. Specific signatures have been associated with lung and/or brain metastasis [21, 22], but their molecular underpinning remain unclear.

Cell lineage specification is molecularly driven by epigenetic mechanisms and in particular chromatin dynamics [23, 24]. Profiling of histone modifications in different mammary cell types reveal a hierarchy of enhancer activation, and unique enhancers present in each mammary lineage can predict transcription factor networks that maintain lineage state [25]. Moreover, enhancer profiling across human breast cancer cell lines identified unique subtype-specific enhancer patterns [26]. It has yet to be determined if particular chromatin alterations can independently explain the metastatic competence or organotropism of breast cancer cells. Furthermore, previous studies have predicted enhancer-driven gene expression changes using a nearest gene approach, whereas experimental techniques like HiChIP enables a more robust way of identifying enhancer-gene links [27, 28]. Also, the transcription factors linked to particular chromatin landscapes and their association with lung or brain metastasis gene expression remain underexplored. Finally, while metastatic signatures have been historically based on gene expression [29], the utility of signatures based on chromatin profiling methods have yet to be evaluated.

Herein we performed an integrated epigenomic analysis using human breast tumors and metastatic breast cancer cells with tropism for lung or brain. By utilizing Chromatin-Immunoprecipitation and sequencing (ChIP-seq) and Assay for Transposase Accessible Chromatin using sequencing (ATAC-seq), we identify substantial changes in the active chromatin landscape of breast cancer cells that are associated with lung and brain metastatic potential. We also incorporate HiChIP data to robustly link enhancers with gene promoters and identify both promoter- and enhancer- driven pathway changes. Finally, we define a chromatin accessibility signature linked to poor patient prognosis and predict transcription factors whose expression or activity within this chromatin state further distinguishes lung and brain metastasis in humans.

## Methods

### Cell lines

MDA-MB-231 (ATCC HTB-26) and its metastatic sub-populations, BrM2 and LM2 have been previously described and were obtained from J. Massagué (Memorial Sloan Kettering Cancer Center, New York) [21, 30]. Cell lines were short tandem repeat tested to be MDA-MB-231 using GenePrint 10 (Promega) and regularly mycoplasma tested (ATCC mycoplasma kit). Cells were grown in DMEM media (Gibco) supplemented with 10% FBS (Gibco) and 1% penicillin (100 U/mL) and streptomycin (100 μg/mL) (Gibco). Cells were grown to 70% confluency prior to all experiments.

### ATAC-seq and data analysis

Omni-ATAC-seq was performed as described in Corces et al. [31]. We generated 2 biological replicates per cell line (harvested at independent passages) and performed paired-end sequencing using an Illumina HiSeq2000 sequencer, averaging 66 million reads per library. Raw reads were trimmed of Nextera adapters using trimmomatic [32] and aligned to hg19 using Bowtie2 [33] with default parameters. Duplicates were marked using picard tools and subsequently bam files were filtered using samtools -F 1804 [34]. To obtain the Tn5 cutsite, forward strand reads were shifted + 5 bp and reverse strand reads were shifted -4 bp. For a consensus ATAC-seq peak set, bam files across cell lines and replicates were first merged together. Peaks were called using MACS2 with parameters –nomodel –keep-dup all -s 1 –shift − 75 –extsize 150 –call-summits [35]. Peaks were then split at their summits using a custom R script. For differential accessibility analysis, reads that fell within the consensus peak set were counted for each replicate of each sample and processed with DESeq2 [36]. Peaks with Benjamini Hochberg (BH) adjusted *p*-value < 0.05 were considered significantly differential. For principal component analysis, averaged variance stabilization transformed read counts of each biological replicate were used. For upset plots comparing the three cell lines, peaks were called within each cell line. Peaks overlapping the consensus ATAC-seq peak set were then used for plotting.

### TCGA ATAC-seq analysis

For meta-analysis of the Corces et al. cohort (*n* = 74), raw counts were downloaded from the breast cancer peak set [37]. Only one primary tumor sample was selected from each patient (*n* = 69). Raw ATAC-seq data from our cell lines were processed through PEPATAC, the same pipeline described in Corces et al. Using the processed bam files, Tn5 cut site reads that fall within the breast cancer peak set were counted by the same process as described in Corces et al. MDA-MB-231 cell line counts were then combined with the counts from the Corces et al. cohort, and the full matrix was normalized using *cpm* with log = TRUE and prior.count = 5 (edgeR package [38]) and *normalize.quantiles* (preprocessCore package [39]).

For the metATAC signature peak set, DESeq2 was first used to determine differential accessibility between parental MDA-MB-231 and both metastatic sub-populations within the aforementioned breast cancer peak set. As we were interested in peaks that were robustly differentially accessible to create our signature, we used an adjusted *p*-value cut-off of 5e-5. For our final parental MDA-MB-231 signal set, we averaged normalized counts falling within the signature peak set between each replicate of parental MDA-MB-231. For our final metastatic signal set, we did the same to all replicates of the metastatic sub-populations. To assign a metATAC score to each patient, we performed a Pearson correlation comparing each patient sample to the parental and metastatic signal sets following the equation:

$$ {S}_{metATAC}={r}_{Met}-{r}_{Par}+1 $$

Where *r*_*Par*_ is the Pearson coefficient for parental signal, *r*_*Met*_ is the coefficient for metastasis signal, and *S*_*metATAC*_ is the metastasis score. Adding 1 was done to ensure positive scores. Therefore, patients whose signature peak set matches more closely to the metastatic lines will have a higher score than patients matching the parental line. For organotropic metATAC scores, we performed the process as explained above, with the modification of performing DESeq2 analysis on the organotropic line versus parental. For the organotropic metastatic signal set, the averaged normalized counts for each replicate of the particular sub-population was used.

### TCGA RNA-seq and survival analysis

RSEM-normalized RNA-seq reads for TCGA patients were downloaded using the R package cgdsr [40] and raw RSEM data were log2 normalized for downstream analyses. PAM50 subtype annotations were retrieved from Netanely et al. [41]. Progression-free interval data were obtained from Liu et al. [42]. For survival analysis, patients were stratified by top and bottom 50th percentiles of metATAC scores.

### RNA-seq and data analysis

Cells were harvested with QIAzol Lysis Reagent (QIAGEN) and homogenized using QIAshredder tubes (QIAGEN). For each cell line, 3 biological replicates were harvested at different passages. RNA isolation was performed using miRNeasy (QIAGEN), and library generation was performed using TruSeq stranded mRNA library prep kit (Illumina). Paired-end sequencing was performed using an Illumina HiSeq4000 sequencer, generating an average of 26 million reads per library. Reads were aligned to hg38 and gene counts to GENCODEv96 transcripts were obtained using STAR aligner v2.7.0 [43] with default parameters. DESeq2 was used to obtain differential gene expression, and HTSFilter [44] was used to filter for expressed genes. Significant differences were identified using a BH adjusted *p*-value cut-off of 0.05.

### ChIP-seq and data analysis

Chromatin Immunoprecipitation and library synthesis were performed as described [45]. Briefly, 10^7^ cells were cross-linked with DMEM media with 1% formaldehyde for 15 min and quenched with 2.5 M glycine. After lysis, nuclei were sonicated with a Bioruptor probe (Diagenode) in an ice water bath set to medium strength in increments of 10 s on and 10 s off for 10 min. For the immunoprecipitation (IP), 5 μg of H3K27ac (Abcam ab4729) or 10 μg of H3K4me3 (Abcam ab8580) antibody was used for each replicate. Library synthesis was performed using the ThruPLEX kit (Takara) and quantified using the NEBNext Library Quant Kit (NEB). Sequencing was performed on an Illumina HiSeq2000 sequencer generating an average of 16 million reads per library. Two biological replicates were generated for each cell line. Reads were aligned and processed as described above. Reads for each IP target were merged and a consensus peak set was called using MACS2 with option –broad. For analyses requiring gene transcription start sites (TSS), TSS data in hg19 coordinates were downloaded from BioMart using the R package biomaRt [46, 47]. For all analyses, only TSS of expressed genes were used. For upset plots comparing the three cell lines, peaks were called within each cell line. Peaks overlapping the respective consensus peak set were then used for plotting.

### HiChIP analysis and gene-enhancer linkages

H3K27ac Hi-ChIP interactions for MDA-MB-231 were first obtained from Cho et al. [48]. All interactions identified in both Hi-ChIP replicates were first assigned to the nearest (within 2000 bp) ATAC-seq consensus peak. Next, only peak-peak interactions that occurred in both Hi-ChIP replicates were kept. ATAC peaks were annotated as enhancers if they overlap with H3K27ac peaks and are greater than 2000 bp away from the nearest TSS, promoters if they overlap H3K4me3 peaks that themselves overlap a TSS, or unknown if they do not satisfy the above criteria. Linkage statistics are enumerated in Supplemental Table 2.

### Differential signal analysis and regulatory motif enrichment

Using consensus peak sets, reads falling within the peaks were counted with featureCounts [49]. Counts were determined for each replicate of each cell line. These replicate counts were then input into DESeq2 [36] for differential binding or accessibility analysis across organotropic (e.g. LM2 versus Par) or metastatic (LM2 and BrM2 versus Par) contrasts. For enrichment of regulatory motifs associated with differentially expressed genes, a motif bed file was first obtained from Marbach et al. [50]. This bed file contained the annotated motif occurrences of 662 TFs across hg19 along with a confidence score based on evolutionary conservation. We first filtered this file for confidence scores greater than 0. Next, we collapsed TFs into clusters based on RSAT matrix clustering of JASPAR CORE vertebrate TFs (http://jaspar.genereg.net/matrix-clusters/vertebrates/) [51]. We named each cluster using the most common TF family within the cluster. To obtain gained or lost motifs, we first counted unique TF clusters occurring under each consensus ATAC-seq peak and then performed hypergeometric tests for each cluster. Significant, differentially accessible ATAC-seq peaks associated with differentially expressed genes (either by promoter or linked enhancers) were used as the foreground and consensus peaks were used as the background. Significant peaks used in the motif analysis were defined as BH adjusted *p*-value < 0.05. *P*-values resulting from the hypergeometric tests were BH adjusted and significance was defined as p-value < 0.05.

### Metastasis-free survival analysis

Because the TCGA cohort does not contain site-specific relapse annotation, we utilized 3 microarray datasets which total 560 patients annotated for site-specific relapse. We downloaded the publicly available datasets MSK and EMC (GSE2603, GSE2034, and GSE12276) [21, 30, 52]. Datasets were RMA normalized and to remove batch effects, expression values were converted to z-scores for all genes across each dataset prior to merging. Annotations were retrieved from Harrell et al. [22]. Relapse and clinical annotations including subtype, hormone receptor status, age, tumor stage, differentiation score, and chemotherapy status were available for 223 patients, and so these patients were used for all Kaplan-Meier (KM) analyses. For all gene-based survival analyses, patients were split at the 50th percentile and top were deemed high while bottom were deemed low. Each gene was represented by its Jetset probe [53]. For single-gene survival correlation, all identified transcription factors belonging to significantly gained or lost motif families were selected for analysis. For each factor, the Cox proportional hazard ratio and *p*-value were calculated using the metastasis-free survival times for relapse to lung, brain, or any site (relapse) and adjusted for covariates subtype, ER, PR, and HER2 statuses, age, tumor stage, differentiation score, and chemotherapy status. Log-rank hazard ratios and *p*-values were also calculated using the log-rank test.

### Statistical analysis and data visualization

All statistical analyses were performed using R version 3.5.2 (R Foundation for Statistical Computing, Vienna, Austria). Heatmaps were generated using deepTools package [54]. Graphs were generated using the R package ggplot2 [55]. Genome track images generated using the R package Gviz [56]. For all Kaplan-Meier plots, log-rank test was used to determine statistical significance. For analysis of multiple intersections, the package UpsetR [57] was used. *P*-values in multiple comparisons were adjusted using BH method.

## Results

### Identifying the chromatin modification and accessibility landscape of metastatic breast cancer cells

To identify epigenomic hallmarks of highly metastatic breast cancer cells, we analyzed the transcriptome and epigenome of cell sub-populations that were in vivo selected from the well-established claudin-low breast cancer cell line MDA-MB-231 (referred to as Par) (Supplemental Table 1). These include LM2–4175 cells (referred to as LM2) [30] and MDA231-BrM2 (referred to as BrM2) [21]. When compared to the Par line, LM2 and BrM2 cells have an enhanced capacity to colonize the lung and brain of mice, respectively. Moreover, these metastatic sub-populations express distinctive gene expression signatures, which do not correlate with detectable protein coding gene mutations [58]. We compared these cell lines using ChIP-seq of active histone marks H3K4me3 and H3K27ac. To determine changes in chromatin accessibility, we also performed ATAC-seq of these cell lines [31]. Putative enhancer regions were defined as distal elements marked with H3K27ac peaks that fall greater than ±2 kb from the nearest transcription start site (TSS) of expressed genes and overlap with an ATAC-seq peak [59, 60]. Predicted promoters, meanwhile, are defined by H3K4me3 peaks falling within ±2 kb from the nearest TSS of expressed genes and overlap with an ATAC-seq peak [60]. Finally, we utilized an MDA-MB-231 HiChIP dataset targeting H3K27ac to identify enhancer-promoter linkages (Supplemental Table 2) [48].

**Fig. 1:**
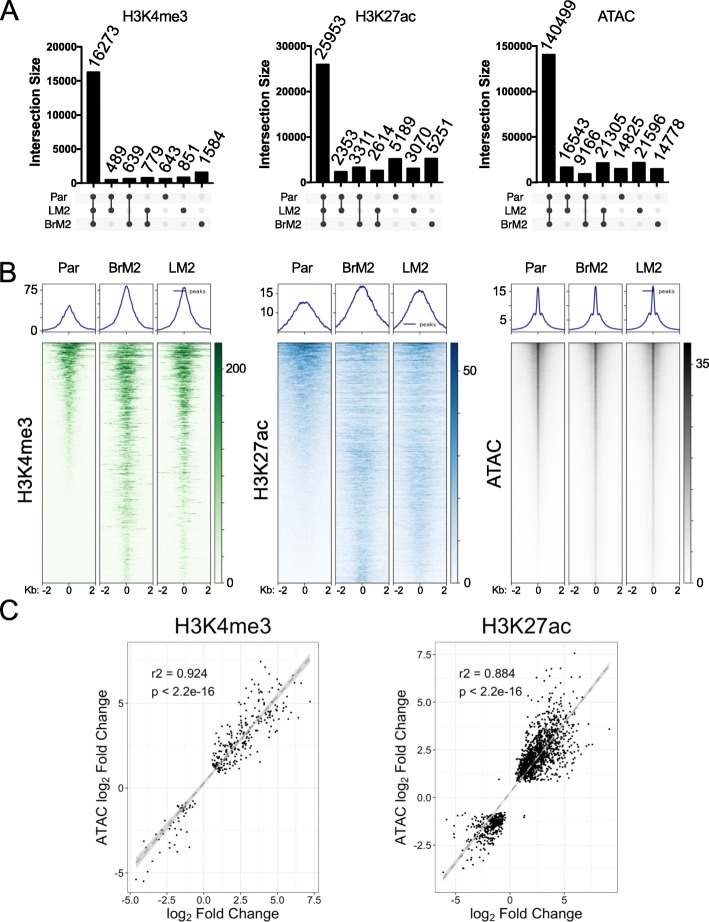
Active epigenetic marks in metastatic breast cancer cells. **a** Upset plots of shared and unique H3K4me3, H3K27ac or ATAC peaks for the indicated cell sub-populations. **b** Heatmaps depicting significantly differential (*p* < 0.05) H3K4me3 (left), H3K27ac (center), and ATAC (right) peaks in the comparison between BrM2 and Par or LM2 and Par. For each mark, genomic regions were ranked based on the signal in Par peaks. *P* values were calculated using DESeq2 and adjusted with the Benjamini-Hochberg (BH) method. **c** Correlation between log2 fold change of each ATAC peak and the log2 fold change of its overlapping H3K4me3 (left) and H3K27ac (right) peak. Log2 fold change is based on the comparison between all metastatic sub-populations and Par cells. Correlation estimate calculated using the Pearson correlation test

Based on histone marks and chromatin accessibility profiles, we found that the metastatic sub-populations harbor epigenomic changes when compared to the less metastatic Par line (Supplemental Table 3). LM2 and BrM2 cells demonstrated distinct H3K4me3, H3K27ac, and open chromatin profiles from Par line. We found that most H3K4me3 (*n* = 16,273), H3K27ac (*n* = 25,953), and ATAC (*n* = 140,499) peaks were shared between the three sub-populations (Fig. 1a). However, there were still specific changes within each metastatic sub-population. Genome-wide heatmaps of significantly altered H3K4me3 peaks (*n* = 1099) show a similar pattern of alterations when comparing both metastatic cell sub-populations to the Par cells (Fig. 1b, left panel). We also found differential H3K27ac (*n* = 3712) peaks and ATAC (*n* = 128,314) peaks (Fig. 1b, middle and right panels) when comparing metastatic sub-populations and Par cells. When comparing both metastatic sub-populations to Par, chromatin accessibility correlated with H3K4me3 and H3K27ac activation (Fig. 1c). We conclude that breast cancer cells harbor significant alterations in active chromatin marks and that these can distinguish metastatic sub-populations from Par cells.

### Brain and lung metastatic competence of breast cancer cells correlates with increased promoter and enhancer activation

Next, we analyzed the molecular consequences of H3K4me3, H3K27ac and chromatin accessibility alterations and their correlations with either multi-organ metastatic potential (alterations which are common to both metastatic sub-populations) versus specific organotropic metastatic predilections to brain or lung.

**Fig. 2:**
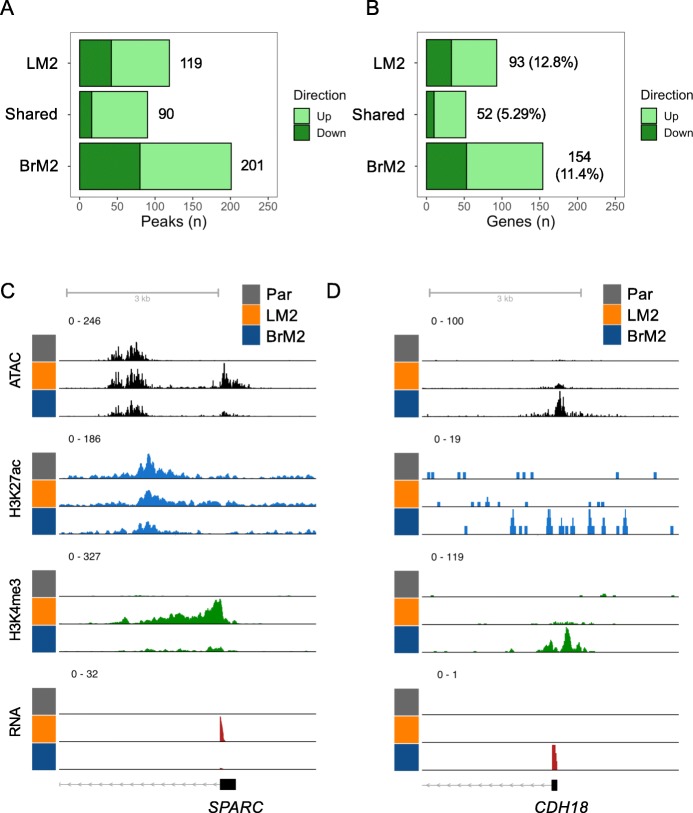
Promoter activation in metastatic cells. **a** Bar plots of the number of significantly decreased (down) and increased (up) H3K4me3 peaks in the indicated comparison. **b** Number of significant concordant gene expression changes associated with significant differential promoter activation plotted as in A. **c** Genome track view of the promoter region of *SPARC*. **d** Genome track view of the promoter region of *CDH18*. All significant peaks defined by *p <* 0.05, as determined by DESeq2 and BH correction. Genome track heights were scaled within each histone modification, ATAC signal, or RNA pile-up

Both LM2 and BrM2 cells displayed significant increases in overall H3K4me3 within annotated promoter regions, with more H3K4me3 peaks associated with either LM2 or BrM2 cells than peaks that are shared by both cell sub-populations (Fig. 2a). To ascertain whether alterations in H3K4me3 are likely to be functional, we integrated our ChIP-seq analysis with our RNA-seq data comparing LM2 and BrM2 with Par cells. The expression of genes with decreased promoter H3K4me3 signal have reduced expression, whereas genes associated with increased H3K4me3 signal have significantly higher expression in LM2 cells or BrM2 compared to Par cells (Figure S1A-B). A small fraction of unique differentially expressed genes was associated with altered promoter activation in LM2 (12.8%) and BrM2 (11.4%) cells, and only 5.29% of shared genes had changes of promoter activation (Fig. 2b). Thus, H3K4me3 changes at the promoters contribute to a small percentage of gene expression changes that confer both overall metastatic potential as well as organotropism. The promoters of several known mediators of metastasis were found to be activated. For example, there was promoter activation of the lung metastasis gene *Secreted Protein Acidic And Cysteine Rich* (*SPARC)* [30] in LM2 cells (Fig. 2c), and brain specific gene *Cadherin 18* (*CDH18*) [61] in BrM2 cells (Fig. 2d). In addition, the promoter H3K4me3 of the *Phospholipase C Beta 1* (*PLCB1)* was similarly activated in LM2 and BrM2 cells (Figure S2A). *PLCB1* has been recently shown to promote breast cancer metastasis [62].

**Fig. 3:**
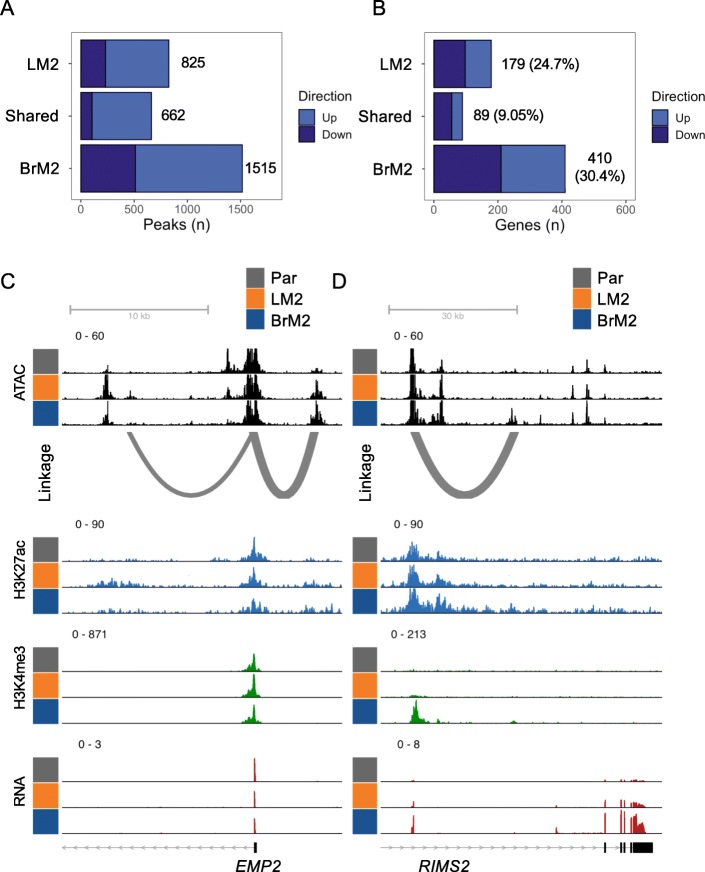
Enhancer activation in metastatic cells. **a** Bar plots of the number of significantly decreased (down) and increased (up) H3K27ac peaks in the indicated comparison. **b** Number of significant gene expression changes associated with significant differential linked enhancer activation plotted as in A. **c** Genome track view of the promoter region of *EMP2* and its promoter-enhancer linkages. **d** Genome track view of the promoter region of *RIMS2* and its promoter-enhancer linkages. All significant peaks defined by *p <* 0.05, as determined by DESeq2 and BH correction. Genome track heights were scaled within each histone modification, ATAC signal, or RNA pile-up

Recent profiling of breast cancer cell lines demonstrated differential enhancer usage across breast cancer subtypes [26]. To characterize changes within putative active enhancers that were associated with metastasis, we focused on H3K27ac regions overlapping ATAC peaks. A general increase in enhancer H3K27ac was observed, especially for BrM2 cells (Fig. 3a). To annotate the genes associated with these putative enhancers, we integrated HiChIP results from MDA-MB-231 cells [48] with our ATAC-seq results to map enhancer-promoter linkages (Supplemental Table 2, methods). As expected, the increased enhancer H3K27ac peaks in metastatic cell sub-populations are associated with increased gene expression, whereas the decreased H3K27ac peaks are linked to decreased gene expression (Figure S1C-D, left). Notably, randomly shuffling the enhancer-promoter linkages abrogated the correlation between H3K27ac signal and gene expression, suggesting that the linkages are biologically relevant (Figure S1C-D, right). 24.7 and 30.4% of unique, differentially expressed genes are associated with enhancer changes in LM2 and BrM2 cells respectively, and only 9.05% of shared genes had changes of enhancer status (Fig. 3b). More than half of the differentially expressed genes are downregulated genes and are associated with decreased H3K27ac in the metastatic cell sub-populations (Fig. 3b). Nonetheless, we found that the enhancers of several known mediators of metastasis are activated. For instance, the enhancer for *Epithelial Membrane Protein-2* (*EMP2*), as shown by HiChIP promoter-enhancer linkage, exhibits increased H3K27ac and ATAC peaks in LM2 cells (Fig. 3c). *EMP2* is implicated in endothelial cell migration [63]. Likewise, the enhancer for *Regulating Synaptic Membrane Exocytosis 2* (*RIMS2*), a gene involved in synaptic neurotransmitter release [64], has increased H3K27ac, H3K4me3, and ATAC peaks in BrM2 cells (Fig. 3d). In addition, the enhancer H3K27ac, H3K4me3 and ATAC levels of *Apolipoprotein B mRNA Editing Enzyme Catalytic Subunit 3G* (*APOBEC3G*) were similarly induced in LM2 and BrM2 cells (Figure S2B). Notably, *APOBEC3G* has been implicated in metastasis of several cancers [65, 66]. Thus, genes involved in overall metastatic potential and organotropism are regulated by chromatin changes at gene enhancers.

Our analysis identifies biologically relevant promoter and enhancer activation states in MDA-MB-231 cell sub-populations which correlate with their overall metastatic potential as well as organotropism for brain or lung.

### A metastasis chromatin accessibility signature associates with human breast cancer subtype and relapse

**Fig. 4:**
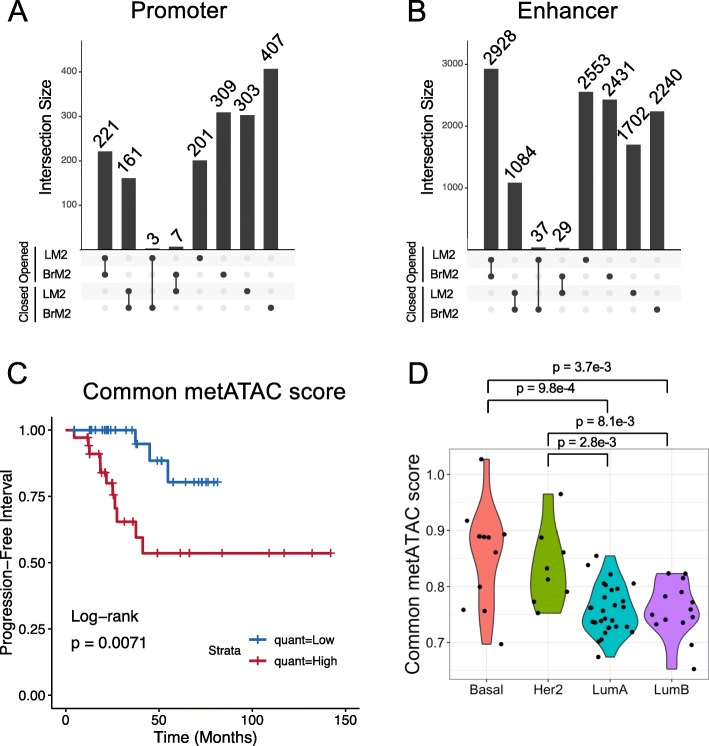
Chromatin accessibility is correlated with breast cancer relapse and subtypes. **a** Upset plot of the shared significant changes (closed or opened) in ATAC-seq peaks that fall within promoter regions between the indicated cell sub-population and Par cells. **b** Upset plot of the shared significant changes (closed or opened) in ATAC-seq peaks that fall within enhancer regions between the indicated cell sub-population and Par cells. **c** Progression free interval of patients stratified by whether their primary tumor common metATAC score was high (above median) or low (below median). *P* value was calculated using log-rank test. **d** Violin plot of common metATAC score of patients separated by PAM50 subtype. *P* values were calculated using Wilcox test

To determine if chromatin accessibility was linked to promoter activation states, we identified the ATAC peaks that overlapped with annotated promoters enriched with H3K4me3. A small proportion of promoter elements with relatively open (*n* = 221) or closed (*n* = 161) chromatin were shared by both metastatic lines, whereas a significant subset of unique promoter regions (*n* = 1220) had altered chromatin accessibility in either LM2 or BrM2 cells alone (Fig. 4a). Similarly, to determine if chromatin accessibility was linked to enhancer activation states, we identified the ATAC-seq regions that overlapped with enhancer-annotated H3K27ac regions. Among the altered enhancers shared by both LM2 and BrM2 cells, 73% (*n* = 2928) of them had increased chromatin accessibility (Fig. 4b). On the other hand, the organotropic chromatin accessibility changes (*n* = 8926) are more equally distributed into increased and decreased categories (Fig. 4b).

We next tested if the chromatin accessibility landscape of metastatic cells was recapitulated in primary breast tumors from human patients with distinct clinical outcome. To this end, we generated a chromatin accessibility signature, which we term the “common metATAC score”, based on ATAC-seq peaks that were significantly altered between all metastatic sub-populations and Par cells (Figure S3A). Primary human breast cancers from The Cancer Genome Atlas (TCGA) were recently profiled using ATAC-seq [37]. We used this data to generate a score based on if their chromatin landscape correlated more (high common metATAC score) or less (low common metATAC score) with that of the metastatic sub-populations. Accordingly, we found that primary tumors with high common metATAC score had a higher probability of relapse (*p* = 0.0071, Fig. 4c). Although site of relapse is not annotated in the TCGA cohort, basal-like and HER2 primary tumors, which are known to be at risk for brain and lung metastasis, had higher common metATAC scores compared to the less metastatic luminal A and B subtypes (Fig. 4d). Because claudin-low was not a part of the official TCGA annotation, we focused on these four main subtypes. GREAT gene ontology analysis showed that several pathways including genes involved in axon midline choice point recognition are associated with increased (up) ATAC peaks in the signature, while pathways including genes involved in protein deubiquitination and response to misfolded protein are correlated with decreased (down) peaks in the signature (Figure S3B). ER status is a well-known early predictor of prognosis and may be a confounder in our survival analysis [67]. When we grouped patients by ER status as determined by IHC, we found a significantly higher metATAC score associated with ER negative patients (Figure S3C). However, the metATAC score is still capable of identifying a subset of ER positive patients with significantly increased risk for relapse (Figure S3D).

These observations demonstrate that shared changes in the chromatin landscape of the highly metastatic MDA-MB-231 cell sub-populations are biologically and clinically relevant.

### Promoter and enhancer driven pathways associated with brain or lung metastasis

**Fig. 5:**
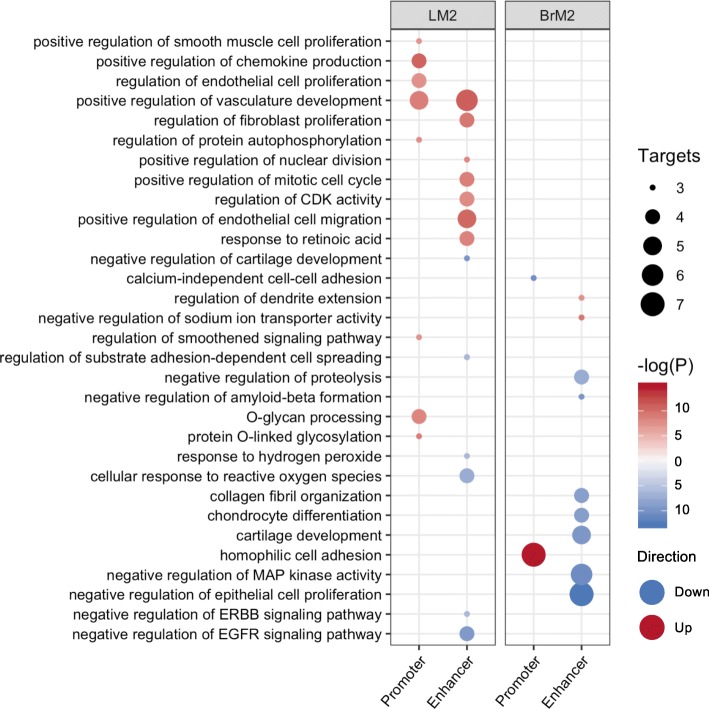
Integrated analyses identify promoter- and enhancer-controlled pathways. EnrichR ontology results of genes differentially regulated by either promoter or enhancer activation. Circle size corresponds to number of terms falling within the corresponding ontology. Color of -log(P) indicates whether the term is gained (red) or lost (blue) in each metastatic sub-population compared to Par

To determine the pathways of the genes associated with promoter and enhancer changes, we integrated the results from ATAC-seq, H3K4me3 and H3K27ac ChIP-seq, RNA-seq and HiChIP linkages for pathway and gene ontology (GO) analysis using EnrichR [68, 69]. For promoter GO we determined genes that are differentially expressed and harbor H3K4me3 changes at their promoter. The same procedure was done for enhancer GO, using HiChIP linkages to determine gene-enhancer associations. These analyses revealed pathways associated with either lung or brain metastasis (Fig. 5, Supplemental Table 4). For example, LM2 enhancer activated genes were enriched for regulators of endothelial cell migration, consistent with the notion that increased migratory ability is a critical property of metastatic cells. LM2 promoter and enhancer activated genes were commonly enriched for regulators of vasculature development, highlighting the importance of vascular remodeling in lung metastasis [70]. BrM2 promoter activated genes were enriched for homophilic cell adhesion, and one example in this pathway is *CDH18*, consistent with the recent reports that cell clusters had higher metastatic potential [71]. On the other hand, enhancer-associated genes in BrM2 cells were mostly associated with suppression of several pathways, including negative regulators of MAP kinase activity and epithelial cell proliferation.

Thus, our integrated analysis identifies biologically relevant pathways associated with organotropism of the MDA-MB-231 cell sub-populations.

### Transcription factors associated with chromatin activation state in metastatic cells

**Fig. 6:**
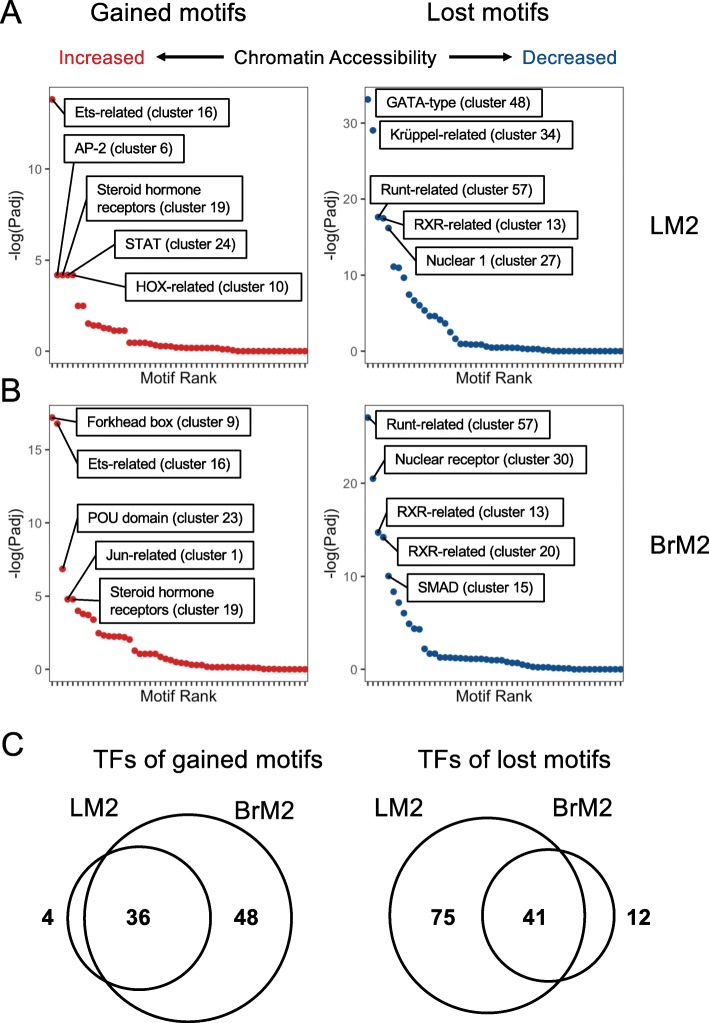
Differentially accessible regions linked to gene expression changes contain specific transcription factor binding motifs. **a**, **b** Transcription factor motifs gained (left) or lost (right) within open-chromatin regions that are linked to differentially expressed genes in LM2 (**a**) or BrM2 (**b**) compared to Par. Significant linkages were determined using HiChIP (see Methods). **c** Venn diagrams of TFs whose motifs are gained (left) or lost (right) in LM2 and BrM2

The high-resolution of differentially open or closed ATAC-seq peaks in the metastatic cells allows us to precisely identify potential transcription factor binding sites. As such, we integrated HiChIP, ATAC-seq and gene expression data to identify motifs of regulatory transcription factors (TFs) that were enriched in chromatin regions with significantly increased (denoted gained TFs) or decreased (denoted lost TFs) accessibility when comparing either LM2 or BrM2 to Par. We determined concordant ATAC peaks linked to gene expression changes between each metastatic sub-population and the Par line, and identified enriched transcription factor motifs in these regions compared to the background of all ATAC peaks (Figure S4, S5, and Fig. 6). Because many transcription factors share similar motifs, we used RSAT clustering to collapse motifs into their respective RSAT cluster [51]. In both the LM2 vs Par and BrM2 vs Par comparisons, motifs for Ets-related, Steroid hormone receptor, and STAT factors were gained, while the Runt-related, Krüppel-related, and nuclear receptor (NR1) motifs were lost (Fig. 6a, b, Figure S5). For lung metastatic LM2 cells, the other significantly gained motifs were AP-2-related and HOX-related factors, while the other top motifs lost include GATA-type and Jun-related factors (Fig. 6a, Figure S5). For brain metastatic BrM2 cells, significantly gained motifs include Forkhead box factors, POU domain, and Jun-related factors, while the top lost motifs are RFX-related, Grainyhead-related, and SMAD factors (Fig. 6b, Figure S5). Motifs for 36 and 41 TFs were gained or lost, respectively, in both metastatic sub-populations (Fig. 6c and Figure S5). On the other hand, LM2 cells specifically gained motifs of 4 TFs and lost motifs of 75 TFs. BrM2 cells have specific gained motifs of 48 TFs, and lost motifs of 12 TFs (Fig. 6c and Figure S5). These analyses suggest that multiple TFs contribute to the shared and distinctive epigenomes of lung and brain metastatic cells.

### Expression of distinct lineage TFs is associated with distal metastasis in human breast cancer patients

The association of certain TF binding motifs with open or closed chromatin regions and transcription in metastatic cells could be due to the aberrant expression of these key lineage TFs. Also, the correlation of the common metATAC score with clinical outcome and breast cancer subtypes suggest that these underlying TFs might be important markers of breast cancer subtype, metastasis to distant organs, or multiple overlapping clinical features.

**Fig. 7:**
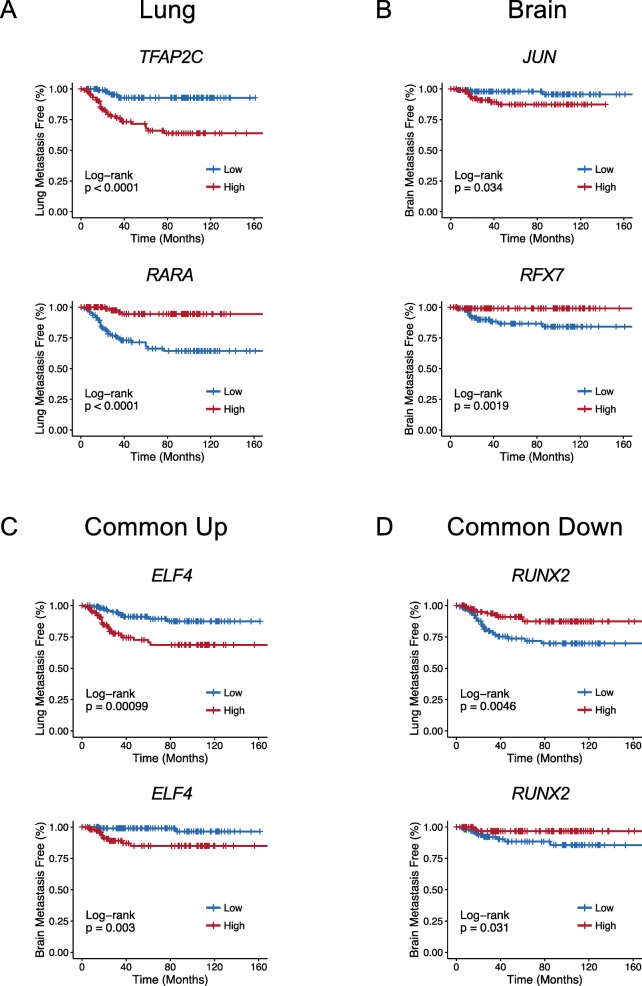
Expression of TFs associated with remodeled chromatin regions correlates with site-specific metastatic relapse. **a**-**d** Examples of Kaplan-Meier curves of TF expression in lung metastasis-free (**a**), brain metastasis-free (**b**), or both lung and brain metastasis-free (**c**, **d**) survival. Human breast tumors from independent institutes were compiled and classified as “high” or “low” based on whether TF expression was above or below the median, respectively. Common up, associated with both lung and brain metastasis; Common down, negatively associated with both lung and brain metastasis. *P* values were calculated by log-rank test. *n* = 223

We first asked whether the TFs associated with the gained or lost motifs correlated with site-specific relapse. In this analysis, we utilized 3 independent breast cancer cohorts (totaling 223 patients after excluding patients with missing clinical covariates) annotated for site-specific relapse [21, 30, 52, 72]. When analyzing the top identified TFs in each organotropic comparison, many known and novel lineage TFs were found to be associated with metastatic relapse to the lung, brain, or both sites using log-rank test or multivariate analyses with Cox Proportional-Hazards (PH) model (Fig. 7 and Supplemental Table 5). For instance, in human primary tumors, high expression of *Transcription Factor AP-2 Gamma* (*TFAP2C*), a TF found to be amplified in 6% of primary breast cancers (cBioPortal [73, 74]), is positively correlated with lung relapse (Fig. 7a, top), while low expression of *Retinoic Acid Receptor Alpha* (*RARA*) is associated with lung relapse (Fig. 7a, bottom). High expression of *JUN* is positively correlated with brain relapse in patients (Fig. 7b, top), while low expression of *Regulatory Factor X7* (*RFX7*) is associated with brain relapse (Fig. 7b, bottom). In addition, high expression of *E74 Like ETS Transcription Factor 4* (*ELF4)* is positively correlated with both lung and brain relapse (Fig. 7c), while low expression of *Runt Related Transcription Factor 2* (also known as *RUNX Family Transcription Factor 2, or RUNX2*) is associated with both lung and brain relapse (Fig. 7d).

Next, we explored the relationship between metastatic chromatin states and their associated TFs with breast cancer subtype and organotropic relapse. The common metATAC score is higher in basal-like and HER2 subtypes (Fig. 4d). The LM2 metATAC score is preferentially increased in basal-like tumors, while BrM2 metATAC score is higher in both basal-like and HER2 subtypes (Figure S6A-B). These results suggest that the chromatin landscape of brain and lung metastatic cells is partially linked to aggressive molecular subtypes. However, among the TFs whose activities are associated with lung or brain metastasis, we found some TFs that were linked to subtype, while others were not. For example, *TFAP2C* is positively associated with lung-specific relapse and expressed at higher levels in basal-like tumors (Fig. 7a and Figure S6C). On the other hand, *RUNX2* is negatively correlated with lung and brain-specific relapse, but is similarly expressed across subtypes (Fig. 7d and Figure S6D). Likewise, *SMAD1* is negatively correlated with brain-specific relapse and expressed at slightly lower levels in basal-like tumors (Figure S6E-F), while *Nuclear Receptor Binding Factor 2* (*NRBF2)* is positively correlated with brain-specific relapse and expressed at similar levels across breast cancer subtypes (Figure S6G-H). Some predicted TFs also exhibit significant correlation with survival within breast cancer subtypes. For instance, *RUNX2* is negatively correlated with lung-specific relapse (*p* = 0.013) and JUN is positively correlated with brain-specific relapse (*p* = 0.0028) within basal-like patients (*n* = 38, Figure S7A-B). This suggests that differential expression of TFs even within subtypes may contribute to increased tumor aggressiveness.

In summary, our approach identifies a chromatin accessibility state in primary breast cancers which is partially linked to intrinsic subtype and increases the likelihood of relapse. Furthermore, this active chromatin state is associated with lineage TFs, whose variable expression may further differentiate brain from lung metastasis.

## Discussion

Several important studies have identified transcriptomic signatures which characterize subgroups of human breast cancers and their response to therapy. Despite these advances, the underlying epigenomic determinants of breast cancer heterogeneity and their influence on metastatic competence are poorly understood. In this study, we integrate epigenomic analysis of the well-established claudin-low MDA-MB-231 breast cancer line and its metastatic lung and brain sub-populations, with recent chromatin accessibility profiling of human breast cancers from TCGA. As the cell model and human datasets characterized herein are widely utilized, our data provides a valuable resource for integrated breast cancer epigenomic studies. Moreover, our findings suggest that chromatin profiling could potentially be leveraged for clinical prognosis, particularly as ATAC-seq can be performed on limited amount of tumor tissue or cells [31].

We identified a chromatin accessibility signature, the common metATAC score, from human tumors which is significantly associated with progression free interval. This finding is consistent with the notion that, in breast cancer, metastatic competence can be enhanced by the epigenetic state of malignant cells within the primary tumor. This active chromatin state is a property of known subtypes of breast cancer, such as the basal-like and HER2 tumors. Nevertheless, we also identified TFs associated with brain or lung relapse for patients independently of subtype (e.g. basal-like). This is in agreement with prior studies that demonstrate that gene expression signatures which correlate with intrinsic subtypes are indicators of distant organ relapse to the lungs and brains, but that additional molecular features of these primary tumors may enhance organotropism [22]. Our results support a model whereby the chromatin landscape of a given breast cancer subtypes predisposes to metastasis, but that the activity of specific TFs further modulates these epigenetic states to influence the site of metastatic colonization.

Differences in promoter and enhancer utilization between primary and metastatic tumors have been documented in several cancer types [75–77]. We find that metastatic breast cancer cell sub-populations from the MDA-MB-231 model generally harbor active promoters and enhancers. When comparing lung and brain metastatic cells to more indolent cells, alterations in promoter activation were more limited whereas activation of enhancers was more widespread. Notably, a significant proportion of differentially regulated enhancers are associated with the modulation of mammary development, morphogenesis, and cell lineage specification [78, 79]. Basal lineage genes are also activated in malignant cells located at the invasive front of primary tumors and are required for dissemination [80]. Thus, tumors with increased metastatic competence may share common epigenomic features linked to lineage subtype (e.g. basal-like). However, significant differences in both promoter and enhancer landscapes could further distinguish tumor cells that preferentially colonize the lung or brain. Importantly, many of these epigenetic alterations were within the regulatory elements of genes that are known to mediate and/or mark lung or brain colonization. It is important to note that only a small proportion of overall gene expression changes is associated with promoter or enhancer histone modification and other layers of epigenetic regulation may play a role in the expression differences between Par and sub-populations.

Ultimately, transcription factors integrate the control of chromatin dynamics with gene expression. In breast cancer, several lineage TFs have been identified as mediators of tumor progression. Although the expression of TFs may be a surrogate for their function, there are many epigenetic processes that ultimately affect their activity [81, 82]. Chromatin accessibility has been successfully used to identify the functional effects of pioneering TFs [83, 84]. Moreover, the technique used in this study, omni-ATAC-seq, has been shown to have the same robustness for TF binding prediction as the previous gold standard DNAase-seq [85]. Our pipeline uncovered robust candidate epigenetic markers of relapse that were not identified by previous reports using ChIP-Seq only [27]. These TFs may help establish or maintain chromatin states which are permissive for the expression of pro-metastatic gene expression programs.

For instance, the presence of AP-2 and Ets motifs gained in metastatic lines demonstrate the presence of canonical active enhancer motifs, as these factors are known to cooperate with lineage TFs to define tissue-specific enhancer usage [86]. Several lineage TFs captured in our analyses are also differentially expressed in various breast cancer subtypes. For instance, the luminal transcription factor *GATA3* is negatively correlated with lung-specific relapse, consistent with the metastasis suppressive role of GATA3. The observation that luminal TFs may be differentially activated in a sub-population of cells from the MDA-MB-231 cells, considered a claudin-low cell line, suggests that plasticity of lineage gene expression can occur within a given subtype. Another group of TFs identified in our study provide novel avenues for exploration. For instance, *TFAP2C* expression was significantly associated with lung relapses, consistent with a recent study [27]. *TFAP2C* is a pioneer factor that plays important roles in pluripotency and lineage differentiation [87, 88]. Interestingly, TFAP2C has been considered to regulate luminal differentiation and its motif is enriched in luminal breast cancer cells [26, 89]. *TFAP2C* overexpression is correlated with shorter survival 10 years after diagnosis and a poorer response to anti-hormone therapy [90, 91]. Finally, we identified transcription factors that commonly correlated with both lung and brain metastasis. This includes *ELF4* which is known for its role in immune response and hematological malignancies [92] but may also regulate functions required for both lung and brain metastasis.

## Conclusions

Integrating RNA-seq, ChIP-seq and ATAC-seq profiling of metastatic cell lines, we identified distinctive epigenomic changes associated with gene pathways in lung and brain metastasis. Using human datasets, we also demonstrated that signatures of metastatic active chromatin are partially linked to breast cancer subtypes with poor prognosis, and that specific TFs are associated with either lung or brain relapse for patients independently of subtype. These findings reveal chromatin states of breast cancers with poor outcome and nominate new potential TF biomarkers for breast cancer metastasis.

## Supplementary information

**Additional file 1: Figure S1.** Gene expression associated with promoter and enhancer changes. (A-B) Distribution of significant gene expression changes (RNA log2FC) associated with promoter H3K4me3 peaks either significantly decreased or increased in LM2 vs Par (A) or BrM2 vs Par (B). (C-D) Distribution of significantly gene expression changes (RNA log2FC) linked with either significantly increased or decreased enhancer H3K27ac peaks in LM2 vs Par (C, left) or BrM2 vs Par (D, left). Linkages determined using HiChIP. Shuffle indicates the same plot after randomly shuffling the linkages. *P* values were calculated using Wilcox U-test. **Figure S2.** Common promoter and enhancer activation in metastatic cells. (A) Genome track view of the promoter region of *PLCB1* demonstrating shared H3K4me3 changes in metastatic sub-populations. (B) Genome track view of *APOBEC3G* demonstrating shared enhancer H3K27ac changes in metastatic sub-populations. Promoter-enhancer linkage was determined by HiChIP. **Figure S3.** metATAC workflow and ontology. (A) Schematic of how the TCGA cohort data and cell line ATAC data were integrated and analyzed. MDA-MB-231 lines were processed using the same method as indicated in Corces et al. [37], and top differentially accessible chromatin regions were used to generate the metATAC signature. Each patient in the TCGA cohort (*n* = 69) was then assigned a score based on their open chromatin similarity to Par or metastatic sub-populations (common metATAC score). Significantly different regions defined by *p* < 5e-5, as determined by DESeq2 and BH correction. (B) GREAT gene ontology results associated with increased (up) and decreased (down) peaks in the signature. (C) Distribution of metATAC score between ER positive and negative patients as determined by IHC. *P*-value determined using Wilcoxon rank sum test. (D) Kaplan-Meier plots of patients separated into ER positive (left) and negative (right) as determined by IHC. metATAC quantile was assigned before splitting the patient into the two groups. **Figure S4.** Scheme for regulatory motif prediction. Scheme for identification of TFs associated with differentially expressed genes between each metastatic sub-population and Par. Reproducible linkages between ATAC peaks were first determined using HiChIP data and subsequently annotated to be either distal or proximal (promoter) to the TSS of each gene. Gene expression is then used to determine significantly up- or down-regulated genes. ATAC peaks that are linked to these gene modules (both distal and proximal) and also follow the same direction of signal change are elected as regulatory regions. Hypergeometric tests are then performed on regulatory regions using all regions as background to determine enriched TF motif clusters (based on RSAT). Motif clusters are finally re-assigned to transcription factors that fall within each respective cluster. **Figure S5.** Regulatory motifs and associated transcription factors. (A) TFs associated with motif clusters enriched in regions of increased accessibility (gained) in either LM2 compared to Par, BrM2 compared to Par, or both. TFs names are color-coded to indicate membership in the respective motif cluster. (B) Same as (A) but with motif clusters enriched in regions of decreased accessibility (lost) in the indicated comparison. **Figure S6.** Subtype specificity of metATAC score and transcription factor expression. (A-B) Distribution of LM2- (A) or BrM2-specific (B) metATAC score across PAM50 subtypes. (C-D) Expression of the indicated transcription factors across subtypes. (E) Kaplan-Meier curve of *SMAD1* expression and brain metastasis-free survival. (F) Expression of *SMAD1* across subtypes. (G) Kaplan-Meier curve of *NRBF2* expression and brain metastasis-free survival. (H) Expression of *NRBF2* across subtypes. NS non-significant. **Figure S7.** Subtype specificity of metATAC score and transcription factor expression. (A) Kaplan-Meier curve of *RUNX2* and lung metastasis-free survival within basal-like patients. (B) Kaplan-Meier curve of *JUN* and brain metastasis-free survival within basal-like patients. *N* = 38.

**Additional file 2: Table S1.** Read count and mapping percentage of all sequenced libraries. **Table S2.** Statistics from HiChIP data. Number and percentage of linked enhancers-promoters and promoters-promoters shown. Genes with linkages are also indicated. **Table S3.** Number of significantly differential peaks and genes in the indicated comparisons.

**Additional file 3: Table S4.** Gene ontologies and pathways. EnrichR results from the analysis of promoter- or enhancer-linked gene expression changes. Each sheet corresponds to a different database that was used as the parameter in the EnrichR analysis.

**Additional file 4: Table S5.** Transcription factor hazard ratio table. Each sheet corresponds to the results from the analysis of organotropic relapse (Lung.relapse and Brain.relapse) or either relapse (Any.relapse). “Notes” sheet includes every column and their descriptions. Transcription factors listed are concordant across two parameters (Hazard ratio by either log-rank or Cox proportional hazard model and the gain/loss of the TF motif). padj Adjusted *P* value; HR Hazard ratio.

## Data Availability

The ChIP-, ATAC-, and RNA-seq datasets generated and analyzed in this study are available in the Gene Expression Omnibus (GEO) repository under the SuperSeries accession number GSE129647 (with SubSeries accessions GSE129645, GSE129646, and GSE138122). We deposited the results from the PEPATAC pipeline applied to our ATAC-seq samples in the SubSeries GSE129646. TCGA gene expression data were retrieved through the cBioPortal R package, cgdsr [40]. Specifically, we used the TCGA Firehose Legacy dataset (caseList parameter: “brca_tcga_all”). The direct download link for this dataset is http://download.cbioportal.org/brca_tcga.tar.gz. PAM50 subtype were retrieved from Ref [41] (Additional file 2), and progression-free survival data from Ref [42] (Table S1). TCGA ATAC-seq data were retrieved from Ref [37] (https://gdc.cancer.gov/about-data/publications/ATACseq-AWG, file: Raw ATAC-seq insertion counts within the pan-cancer peak set). For metastasis-free survival analysis, datasets GSE2603, GSE2034, and GSE12276 [21, 30, 52] were used. MDA-MB-231 HiChIP data were obtained from [48] (GSE97585). R scripts are deposited in https://github.com/wesleylcai/bmcmedgenomics2020_metastasis.

## Abbreviations

APOBEC3G: Apolipoprotein B mRNA Editing Enzyme, Catalytic Polypeptide-like 3G
ATAC-seq: Assay for Transposase-Accessible Chromatin using sequencing
BH: Benjamini-Hochberg
bp: Base pair
BrM2: MDA231-BrM2 cell line
CDH18: Cadherin 18
ChIP-seq: Chromatin Immunoprecipitation and sequencing
ELF4: E74 Like ETS Transcription Factor 4
EMP2: Epithelial Membrane Protein 2
ER: Estrogen receptor
KM: Kaplan-Meier
LM2: LM2–4175 cell line
NRBF2: Nuclear Receptor Binding Factor 2
Par: MDA-MB-231 cell line
PLCB1: Phospholipase C Beta 1
PR: Progesterone receptor
RARA: Retinoic Acid Receptor Alpha
RFX7: Regulatory Factor X7
RIMS2: Regulating Synaptic Membrane Exocytosis 2
RUNX2: RUNX Family Transcription Factor 2
SMAD1: SMAD Family Member 1
SPARC: Secreted Protein Acidic And Cysteine Rich
TCGA: The Cancer Genome Atlas
TF: Transcription factor
TFAP2C: Transcription Factor AP-2 Gamma
TSS: Transcription start site

## References

[1] Siegel RL, Miller KD, Jemal A (2019). Cancer statistics, 2019. CA Cancer J Clin.

[2] Torre LA, Islami F, Siegel RL, Ward EM, Jemal A (2017). Global Cancer in women: burden and trends. Cancer Epidemiol Biomark Prev.

[3] Perou CM, Sorlie T, Eisen MB, van de Rijn M, Jeffrey SS, Rees CA, Pollack JR, Ross DT, Johnsen H, Akslen LA (2000). Molecular portraits of human breast tumours. Nature.

[4] Herschkowitz JI, Simin K, Weigman VJ, Mikaelian I, Usary J, Hu Z, Rasmussen KE, Jones LP, Assefnia S, Chandrasekharan S (2007). Identification of conserved gene expression features between murine mammary carcinoma models and human breast tumors. Genome Biol.

[5] Prat A, Parker JS, Karginova O, Fan C, Livasy C, Herschkowitz JI, He X, Perou CM (2010). Phenotypic and molecular characterization of the claudin-low intrinsic subtype of breast cancer. Breast Cancer Res.

[6] Ohara AM, Naoi Y, Shimazu K, Kagara N, Shimoda M, Tanei T, Miyake T, Kim SJ, Noguchi S (2019). PAM50 for prediction of response to neoadjuvant chemotherapy for ER-positive breast cancer. Breast Cancer Res Treat.

[7] Laenkholm AV, Jensen MB, Eriksen JO, Rasmussen BB, Knoop AS, Buckingham W, Ferree S, Schaper C, Haffner T, Nielsen TO (2018). PAM50 risk of recurrence score predicts 10-year distant recurrence in a comprehensive Danish cohort of postmenopausal women allocated to 5 years of endocrine therapy for hormone receptor-positive early breast Cancer. J Clin Oncol.

[8] Allott EH, Geradts J, Cohen SM, Khoury T, Zirpoli GR, Bshara W, Davis W, Omilian A, Nair P, Ondracek RP (2018). Frequency of breast cancer subtypes among African American women in the AMBER consortium. Breast Cancer Res.

[9] Holm J, Eriksson L, Ploner A, Eriksson M, Rantalainen M, Li J, Hall P, Czene K (2017). Assessment of breast Cancer risk factors reveals subtype heterogeneity. Cancer Res.

[10] Visvader JE, Stingl J (2014). Mammary stem cells and the differentiation hierarchy: current status and perspectives. Genes Dev.

[11] Raouf A, Zhao Y, Stingl J, Delaney A, Barbara M, Iscove N, Jones S, McKinney S, Emerman J, To K (2008). Transcriptome analysis of the normal human mammary cell commitment and differentiation process. Cell Stem Cell.

[12] Kumar B, Prasad M, Bhat-Nakshatri P, Anjanappa M, Kalra M, Marino N, Storniolo AM, Rao X, Liu S, Wan J (2018). Normal breast-derived epithelial cells with luminal and intrinsic subtype-enriched gene expression document Interindividual differences in their differentiation Cascade. Cancer Res.

[13] Nguyen DX, Bos PD, Massague J (2009). Metastasis: from dissemination to organ-specific colonization. Nat Rev Cancer.

[14] Medeiros Braeden, Allan Alison L. (2019). Molecular Mechanisms of Breast Cancer Metastasis to the Lung: Clinical and Experimental Perspectives. International Journal of Molecular Sciences.

[15] Leone JP, Leone BA (2015). Breast cancer brain metastases: the last frontier. Exp Hematol Oncol.

[16] Schrijver W, Selenica P, Lee JY, Ng CKY, Burke KA, Piscuoglio S, Berman SH, Reis-Filho JS, Weigelt B, van Diest PJ (2018). Mutation profiling of key Cancer genes in primary breast cancers and their distant metastases. Cancer Res.

[17] Chen W, Hoffmann AD, Liu H, Liu X (2018). Organotropism: new insights into molecular mechanisms of breast cancer metastasis. NPJ Precis Oncol.

[18] Bertucci F, Finetti P, Birnbaum D (2012). Basal breast cancer: a complex and deadly molecular subtype. Curr Mol Med.

[19] Lassman AB, DeAngelis LM (2003). Brain metastases. Neurol Clin.

[20] Slimane K, Andre F, Delaloge S, Dunant A, Perez A, Grenier J, Massard C, Spielmann M (2004). Risk factors for brain relapse in patients with metastatic breast cancer. Ann Oncol.

[21] Bos PD, Zhang XH, Nadal C, Shu W, Gomis RR, Nguyen DX, Minn AJ, van de Vijver MJ, Gerald WL, Foekens JA (2009). Genes that mediate breast cancer metastasis to the brain. Nature.

[22] Harrell JC, Prat A, Parker JS, Fan C, He X, Carey L, Anders C, Ewend M, Perou CM (2012). Genomic analysis identifies unique signatures predictive of brain, lung, and liver relapse. Breast Cancer Res Treat.

[23] Chen T, Dent SY (2014). Chromatin modifiers and remodellers: regulators of cellular differentiation. Nat Rev Genet.

[24] Atlasi Y, Stunnenberg HG (2017). The interplay of epigenetic marks during stem cell differentiation and development. Nat Rev Genet.

[25] Pellacani D, Tan S, Lefort S, Eaves CJ (2019). Transcriptional regulation of normal human mammary cell heterogeneity and its perturbation in breast cancer. EMBO J.

[26] Franco HL, Nagari A, Malladi VS, Li W, Xi Y, Richardson D, Allton KL, Tanaka K, Li J, Murakami S (2018). Enhancer transcription reveals subtype-specific gene expression programs controlling breast cancer pathogenesis. Genome Res.

[27] Li K, Xu C, Du Y, Junaid M, Kaushik AC, Wei DQ (2019). Comprehensive epigenetic analyses reveal master regulators driving lung metastasis of breast cancer. J Cell Mol Med.

[28] Mumbach MR, Rubin AJ, Flynn RA, Dai C, Khavari PA, Greenleaf WJ, Chang HY (2016). HiChIP: efficient and sensitive analysis of protein-directed genome architecture. Nat Methods.

[29] Smith HA, Kang Y (2017). Determinants of Organotropic metastasis. Ann Rev Cancer Biol.

[30] Minn AJ, Gupta GP, Siegel PM, Bos PD, Shu W, Giri DD, Viale A, Olshen AB, Gerald WL, Massague J (2005). Genes that mediate breast cancer metastasis to lung. Nature.

[31] Corces MR, Trevino AE, Hamilton EG, Greenside PG, Sinnott-Armstrong NA, Vesuna S, Satpathy AT, Rubin AJ, Montine KS, Wu B (2017). An improved ATAC-seq protocol reduces background and enables interrogation of frozen tissues. Nat Methods.

[32] Bolger AM, Lohse M, Usadel B (2014). Trimmomatic: a flexible trimmer for Illumina sequence data. Bioinformatics.

[33] Langmead B, Salzberg SL (2012). Fast gapped-read alignment with bowtie 2. Nat Methods.

[34] Li H, Handsaker B, Wysoker A, Fennell T, Ruan J, Homer N, Marth G, Abecasis G, Durbin R (2009). Genome project data processing S: the sequence alignment/map format and SAMtools. Bioinformatics.

[35] Zhang Y, Liu T, Meyer CA, Eeckhoute J, Johnson DS, Bernstein BE, Nusbaum C, Myers RM, Brown M, Li W (2008). Model-based analysis of ChIP-Seq (MACS). Genome Biol.

[36] Love MI, Huber W, Anders S (2014). Moderated estimation of fold change and dispersion for RNA-seq data with DESeq2. Genome Biol.

[37] Corces M. Ryan, Granja Jeffrey M., Shams Shadi, Louie Bryan H., Seoane Jose A., Zhou Wanding, Silva Tiago C., Groeneveld Clarice, Wong Christopher K., Cho Seung Woo, Satpathy Ansuman T., Mumbach Maxwell R., Hoadley Katherine A., Robertson A. Gordon, Sheffield Nathan C., Felau Ina, Castro Mauro A. A., Berman Benjamin P., Staudt Louis M., Zenklusen Jean C., Laird Peter W., Curtis Christina, Greenleaf William J., Chang Howard Y. (2018). The chromatin accessibility landscape of primary human cancers. Science.

[38] Robinson MD, McCarthy DJ, Smyth GK (2010). edgeR: a bioconductor package for differential expression analysis of digital gene expression data. Bioinformatics.

[39] preprocessCore: A collection of pre-processing functions [https://github.com/bmbolstad/preprocessCore].

[40] cgdsr: R-Based API for Accessing the MSKCC Cancer Genomics Data Server (CGDS) [https://CRAN.R-project.org/package=cgdsr].

[41] Netanely D, Avraham A, Ben-Baruch A, Evron E, Shamir R (2016). Expression and methylation patterns partition luminal-a breast tumors into distinct prognostic subgroups. Breast Cancer Res.

[42] Liu J, Lichtenberg T, Hoadley KA, Poisson LM, Lazar AJ, Cherniack AD, Kovatich AJ, Benz CC, Levine DA, Lee AV (2018). An integrated TCGA pan-Cancer clinical data resource to drive high-quality survival outcome analytics. Cell.

[43] Dobin A, Davis CA, Schlesinger F, Drenkow J, Zaleski C, Jha S, Batut P, Chaisson M, Gingeras TR (2013). STAR: ultrafast universal RNA-seq aligner. Bioinformatics.

[44] Rau A, Gallopin M, Celeux G, Jaffrezic F (2013). Data-based filtering for replicated high-throughput transcriptome sequencing experiments. Bioinformatics.

[45] Greer CB, Tanaka Y, Kim YJ, Xie P, Zhang MQ, Park IH, Kim TH (2015). Histone Deacetylases positively regulate transcription through the elongation machinery. Cell Rep.

[46] Durinck S, Moreau Y, Kasprzyk A, Davis S, De Moor B, Brazma A, Huber W (2005). BioMart and bioconductor: a powerful link between biological databases and microarray data analysis. Bioinformatics.

[47] Durinck S, Spellman PT, Birney E, Huber W (2009). Mapping identifiers for the integration of genomic datasets with the R/bioconductor package biomaRt. Nat Protoc.

[48] Cho SW, Xu J, Sun R, Mumbach MR, Carter AC, Chen YG, Yost KE, Kim J, He J, Nevins SA (2018). Promoter of lncRNA gene PVT1 is a tumor-suppressor DNA boundary element. Cell.

[49] Liao Y, Smyth GK, Shi W (2014). FeatureCounts: an efficient general purpose program for assigning sequence reads to genomic features. Bioinformatics.

[50] Marbach D, Lamparter D, Quon G, Kellis M, Kutalik Z, Bergmann S (2016). Tissue-specific regulatory circuits reveal variable modular perturbations across complex diseases. Nat Methods.

[51] Castro-Mondragon JA, Jaeger S, Thieffry D, Thomas-Chollier M, van Helden J (2017). RSAT matrix-clustering: dynamic exploration and redundancy reduction of transcription factor binding motif collections. Nucleic Acids Res.

[52] Wang Y, Klijn JG, Zhang Y, Sieuwerts AM, Look MP, Yang F, Talantov D, Timmermans M, Meijer-van Gelder ME, Yu J (2005). Gene-expression profiles to predict distant metastasis of lymph-node-negative primary breast cancer. Lancet.

[53] Li Q, Birkbak NJ, Gyorffy B, Szallasi Z, Eklund AC (2011). Jetset: selecting the optimal microarray probe set to represent a gene. BMC Bioinformatics.

[54] Ramirez F, Dundar F, Diehl S, Gruning BA, Manke T (2014). deepTools: a flexible platform for exploring deep-sequencing data. Nucleic Acids Res.

[55] Wickham H (2016). ggplot2: Elegant Graphics for Data Analysis.

[56] Hahne F, Ivanek R (2016). Visualizing genomic data using Gviz and bioconductor. Methods Mol Biol.

[57] Conway JR, Lex A, Gehlenborg N (2017). UpSetR: an R package for the visualization of intersecting sets and their properties. Bioinformatics.

[58] Jacob LS, Vanharanta S, Obenauf AC, Pirun M, Viale A, Socci ND, Massague J (2015). Metastatic competence can emerge with selection of preexisting oncogenic alleles without a need of new mutations. Cancer Res.

[59] Heintzman ND, Hon GC, Hawkins RD, Kheradpour P, Stark A, Harp LF, Ye Z, Lee LK, Stuart RK, Ching CW (2009). Histone modifications at human enhancers reflect global cell-type-specific gene expression. Nature.

[60] Ernst J, Kheradpour P, Mikkelsen TS, Shoresh N, Ward LD, Epstein CB, Zhang X, Wang L, Issner R, Coyne M (2011). Mapping and analysis of chromatin state dynamics in nine human cell types. Nature.

[61] Shibata Tatsuhiro, Shimoyama Yutaka, Gotoh Masahiro, Hirohashi Setsuo (1997). Identification of Human Cadherin-14, a Novel Neurally Specific Type II Cadherin, by Protein Interaction Cloning. Journal of Biological Chemistry.

[62] Sengelaub CA, Navrazhina K, Ross JB, Halberg N, Tavazoie SF (2016). PTPRN2 and PLCbeta1 promote metastatic breast cancer cell migration through PI(4,5)P2-dependent actin remodeling. EMBO J.

[63] Gordon LK, Kiyohara M, Fu M, Braun J, Dhawan P, Chan A, Goodglick L, Wadehra M (2013). EMP2 regulates angiogenesis in endometrial cancer cells through induction of VEGF. Oncogene.

[64] Wang Y, Sudhof TC (2003). Genomic definition of RIM proteins: evolutionary amplification of a family of synaptic regulatory proteins. Genomics.

[65] Ding Q, Chang CJ, Xie X, Xia W, Yang JY, Wang SC, Wang Y, Xia J, Chen L, Cai C (2011). APOBEC3G promotes liver metastasis in an orthotopic mouse model of colorectal cancer and predicts human hepatic metastasis. J Clin Invest.

[66] Lan H, Jin K, Gan M, Wen S, Bi T, Zhou S, Zhu N, Teng L, Yu W (2014). APOBEC3G expression is correlated with poor prognosis in colon carcinoma patients with hepatic metastasis. Int J Clin Exp Med.

[67] Bentzon N, During M, Rasmussen BB, Mouridsen H, Kroman N (2008). Prognostic effect of estrogen receptor status across age in primary breast cancer. Int J Cancer.

[68] Kuleshov MV, Jones MR, Rouillard AD, Fernandez NF, Duan Q, Wang Z, Koplev S, Jenkins SL, Jagodnik KM, Lachmann A (2016). Enrichr: a comprehensive gene set enrichment analysis web server 2016 update. Nucleic Acids Res.

[69] Chen EY, Tan CM, Kou Y, Duan Q, Wang Z, Meirelles GV, Clark NR, Ma'ayan A (2013). Enrichr: interactive and collaborative HTML5 gene list enrichment analysis tool. BMC Bioinformatics.

[70] Gupta GP, Nguyen DX, Chiang AC, Bos PD, Kim JY, Nadal C, Gomis RR, Manova-Todorova K, Massague J (2007). Mediators of vascular remodelling co-opted for sequential steps in lung metastasis. Nature.

[71] Gkountela S, Castro-Giner F, Szczerba BM, Vetter M, Landin J, Scherrer R, Krol I, Scheidmann MC, Beisel C, Stirnimann CU (2019). Circulating tumor cell clustering shapes DNA methylation to enable metastasis seeding. Cell.

[72] Minn AJ, Gupta GP, Padua D, Bos P, Nguyen DX, Nuyten D, Kreike B, Zhang Y, Wang Y, Ishwaran H (2007). Lung metastasis genes couple breast tumor size and metastatic spread. Proc Natl Acad Sci U S A.

[73] Cerami E, Gao J, Dogrusoz U, Gross BE, Sumer SO, Aksoy BA, Jacobsen A, Byrne CJ, Heuer ML, Larsson E (2012). The cBio cancer genomics portal: an open platform for exploring multidimensional cancer genomics data. Cancer Discov.

[74] Gao J, Aksoy BA, Dogrusoz U, Dresdner G, Gross B, Sumer SO, Sun Y, Jacobsen A, Sinha R, Larsson E (2013). Integrative analysis of complex cancer genomics and clinical profiles using the cBioPortal. Sci Signal.

[75] Denny SK, Yang D, Chuang CH, Brady JJ, Lim JS, Gruner BM, Chiou SH, Schep AN, Baral J, Hamard C (2016). Nfib promotes metastasis through a widespread increase in chromatin accessibility. Cell.

[76] Roe JS, Hwang CI, Somerville TDD, Milazzo JP, Lee EJ, Da Silva B, Maiorino L, Tiriac H, Young CM, Miyabayashi K (2017). Enhancer reprogramming promotes pancreatic Cancer metastasis. Cell.

[77] Morrow JJ, Bayles I, Funnell APW, Miller TE, Saiakhova A, Lizardo MM, Bartels CF, Kapteijn MY, Hung S, Mendoza A (2018). Positively selected enhancer elements endow osteosarcoma cells with metastatic competence. Nat Med.

[78] Lee HK, Willi M, Shin HY, Liu C, Hennighausen L (2018). Progressing super-enhancer landscape during mammary differentiation controls tissue-specific gene regulation. Nucleic Acids Res.

[79] Pellacani D, Bilenky M, Kannan N, Heravi-Moussavi A, Knapp D, Gakkhar S, Moksa M, Carles A, Moore R, Mungall AJ (2016). Analysis of Normal human mammary Epigenomes reveals cell-specific active enhancer states and associated transcription factor networks. Cell Rep.

[80] Cheung KJ, Gabrielson E, Werb Z, Ewald AJ (2013). Collective invasion in breast cancer requires a conserved basal epithelial program. Cell.

[81] Filtz TM, Vogel WK, Leid M (2014). Regulation of transcription factor activity by interconnected post-translational modifications. Trends Pharmacol Sci.

[82] Tootle TL, Rebay I (2005). Post-translational modifications influence transcription factor activity: a view from the ETS superfamily. Bioessays.

[83] Pique-Regi R, Degner JF, Pai AA, Gaffney DJ, Gilad Y, Pritchard JK (2011). Accurate inference of transcription factor binding from DNA sequence and chromatin accessibility data. Genome Res.

[84] Sherwood RI, Hashimoto T, O'Donnell CW, Lewis S, Barkal AA, van Hoff JP, Karun V, Jaakkola T, Gifford DK (2014). Discovery of directional and nondirectional pioneer transcription factors by modeling DNase profile magnitude and shape. Nat Biotechnol.

[85] Li Z, Schulz MH, Look T, Begemann M, Zenke M, Costa IG (2019). Identification of transcription factor binding sites using ATAC-seq. Genome Biol.

[86] Vierbuchen T, Ling E, Cowley CJ, Couch CH, Wang X, Harmin DA, Roberts CWM, Greenberg ME (2017). AP-1 transcription factors and the BAF complex mediate signal-dependent enhancer selection. Mol Cell.

[87] Li L, Wang Y, Torkelson JL, Shankar G, Pattison JM, Zhen HH, Fang F, Duren Z, Xin J, Gaddam S (2019). TFAP2C- and p63-dependent networks sequentially rearrange chromatin landscapes to drive human epidermal lineage commitment. Cell Stem Cell.

[88] Pastor WA, Liu W, Chen D, Ho J, Kim R, Hunt TJ, Lukianchikov A, Liu X, Polo JM, Jacobsen SE (2018). TFAP2C regulates transcription in human naive pluripotency by opening enhancers. Nat Cell Biol.

[89] Cyr AR, Kulak MV, Park JM, Bogachek MV, Spanheimer PM, Woodfield GW, White-Baer LS, O'Malley YQ, Sugg SL, Olivier AK (2015). TFAP2C governs the luminal epithelial phenotype in mammary development and carcinogenesis. Oncogene.

[90] Perkins SM, Bales C, Vladislav T, Althouse S, Miller KD, Sandusky G, Badve S, Nakshatri H (2015). TFAP2C expression in breast cancer: correlation with overall survival beyond 10 years of initial diagnosis. Breast Cancer Res Treat.

[91] Gee JM, Eloranta JJ, Ibbitt JC, Robertson JF, Ellis IO, Williams T, Nicholson RI, Hurst HC (2009). Overexpression of TFAP2C in invasive breast cancer correlates with a poorer response to anti-hormone therapy and reduced patient survival. J Pathol.

[92] Suico MA, Shuto T, Kai H (2017). Roles and regulations of the ETS transcription factor ELF4/MEF. J Mol Cell Biol.

