# Supplementary material for: Specific chromatin landscapes and transcription factors couple breast cancer subtype with metastatic relapse to lung or brain
Source: BMC Med Genomics. 2020 Mar 6;13:33. doi: 10.1186/s12920-020-0695-0 (PMC7060551; doi:10.1186/s12920-020-0695-0)
Supplement: Supplementary file 1 — Additional file 1: Figure S1. Gene expression associated with promoter and enhancer changes. (A-B) Distribution of significant gene expression changes (RNA log2FC) associated with promoter H3K4me3 peaks either significantly decreased or increased in LM2 vs Par (A) or BrM2 vs Par (B). (C-D) Distribution of significantly gene expression changes (RNA log2FC) linked with either significantly increased or decreased enhancer H3K27ac peaks in LM2 vs Par (C, left) or BrM2 vs Par (D, left). Linkages determined using HiChIP. Shuffle indicates the same plot after randomly shuffling the linkages. P values were calculated using Wilcox U-test. Figure S2. Common promoter and enhancer activation in metastatic cells. (A) Genome track view of the promoter region of PLCB1 demonstrating shared H3K4me3 changes in metastatic sub-populations. (B) Genome track view of APOBEC3G demonstrating shared enhancer H3K27ac changes in metastatic sub-populations. Promoter-enhancer linkage was determined by HiChIP. Figure S3. metATAC workflow and ontology. (A) Schematic of how the TCGA cohort data and cell line ATAC data were integrated and analyzed. MDA-MB-231 lines were processed using the same method as indicated in Corces et al. [37], and top differentially accessible chromatin regions were used to generate the metATAC signature. Each patient in the TCGA cohort (n = 69) was then assigned a score based on their open chromatin similarity to Par or metastatic sub-populations (common metATAC score). Significantly different regions defined by p < 5e-5, as determined by DESeq2 and BH correction. (B) GREAT gene ontology results associated with increased (up) and decreased (down) peaks in the signature. (C) Distribution of metATAC score between ER positive and negative patients as determined by IHC. P-value determined using Wilcoxon rank sum test. (D) Kaplan-Meier plots of patients separated into ER positive (left) and negative (right) as determined by IHC. metATAC quantile was assigned before splitting t [file 12920_2020_695_MOESM1_ESM.pdf]

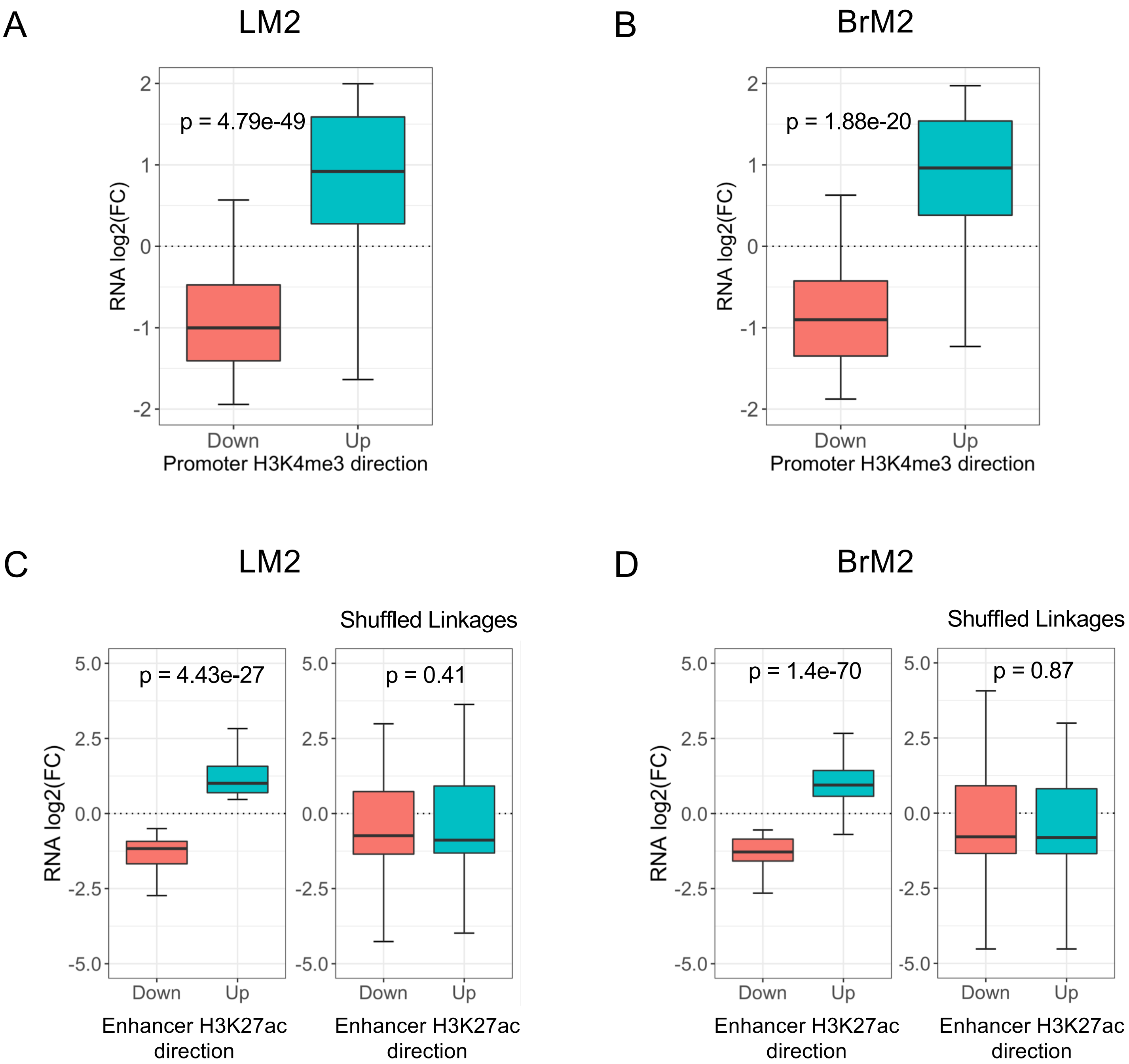

Supplemental Figure 1

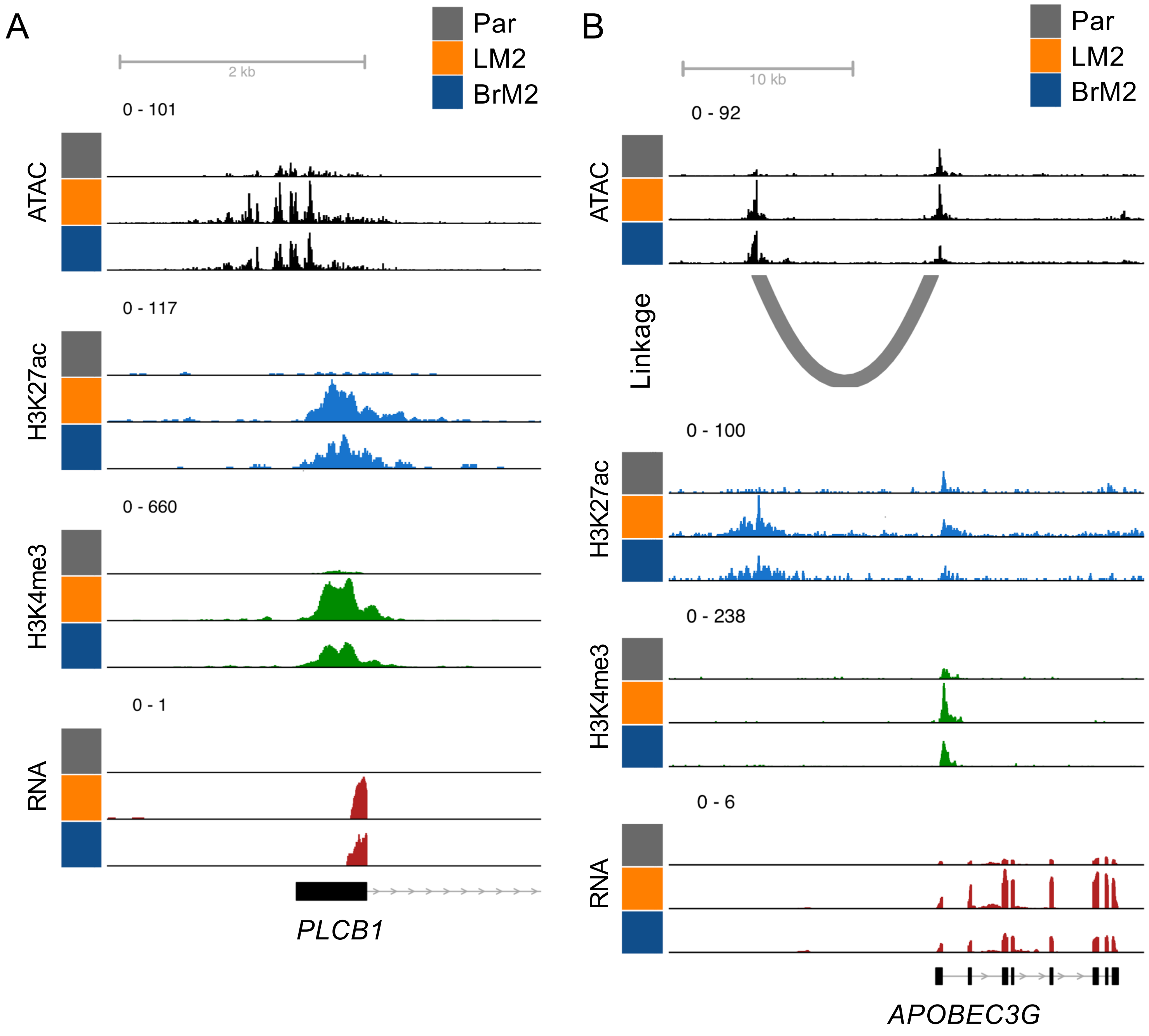

Supplemental Figure 2

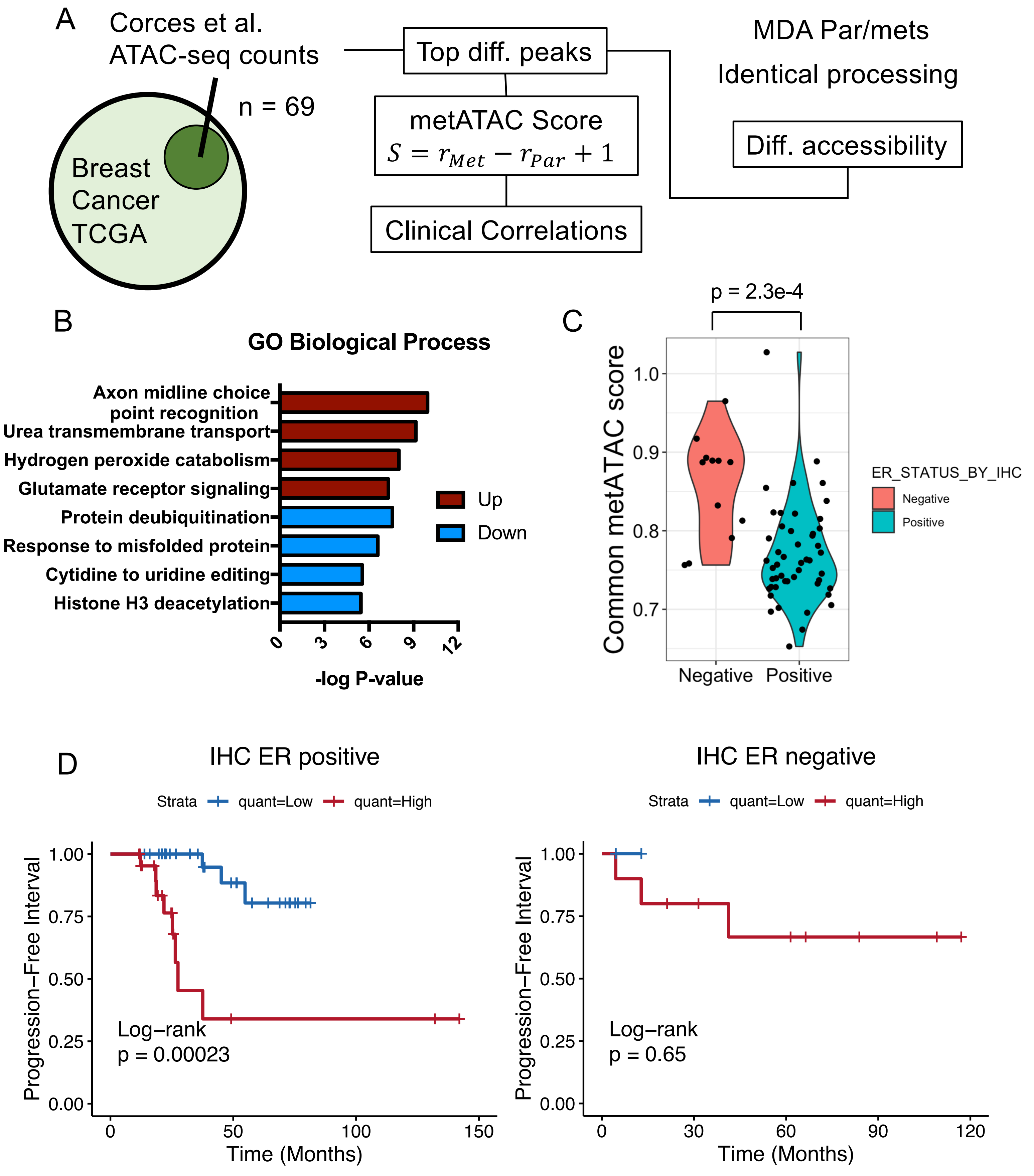

Supplemental Figure 3

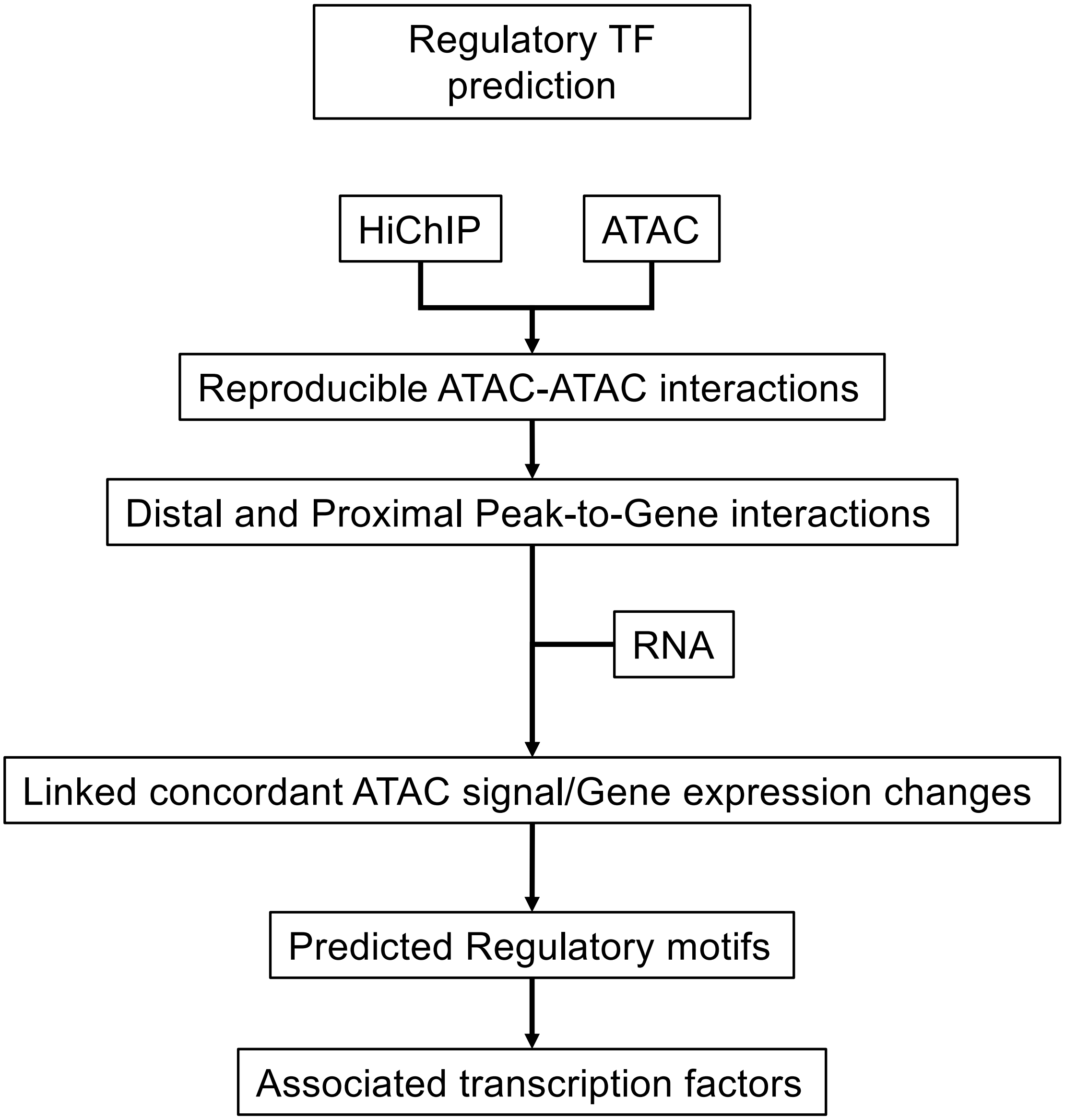

Supplemental Figure 4

A

LM2 Gained	Shared Gained		BrM2 Gained			TF Cluster
TFAP2A	EHF	AR	FOXA1	BACH1	ATF4	AP-2_cluster_6
TFAP2C	ELF1	NR3C1	FOXA2	BACH2	CEBPB	HOX-related factors_cluster_10
TFAP2E	ELF2	NR3C2	FOXA3	BATF	CEBPD	Ets-related factors_cluster_16
HOXB9	ELF3	BCL6	FOXC1	BATF3	CEBPG	Steroid hormone (NR3)_cluster_19
	ELF4	NFAT5	FOXF2	FOS	DBP	STAT factors_cluster_24
	ELK1	NFATC1	FOXJ2	FOSL1	NFIL3	Forkhead box (FOX) factors_cluster_9
	ELK3	NFATC2	FOXJ3	FOSL2	TEF	POU domain factors_cluster_23
	ELK4	NFATC3	FOXK1	JDP2		Jun-related factors_cluster_1
	ERF	NFATC4	FOXK2	JUN		TEF-1-related factors_cluster_2
	ETS1	RBPJ	FOXN3	JUNB		C/EBP-related_cluster_5
	ETS2	STAT1	FOXO1	JUND		
	ETV1	STAT3	FOXO3	MAFK		
	ETV3	STAT5A	FOXO4	NFE2L1		
	ETV4	STAT5B	FOXO6	NFE2L2		
	ETV5	STAT6	FOXP1	HNF4G		
	ETV6	ZNF75D	FOXP2	TEAD1		
	GABPA		FOXQ1	TEAD2		
	SPDEF		LIN54	TEAD3		
	ZBTB7A		POU2F1	TEAD4		
	ZKSCAN5		POU2F2	ZNF684		
			POU3F2			

B

LM2 Lost				Shared Lost		BrM2 Lost	TF Cluster
GATA2	BACH1	ARNT	MNT	RUNX1	RARA	SMAD1	GATA-type zinc fingers_cluster_48
GATA3	BACH2	ARNT2	MYC	RUNX2	RARB	SMAD3	Nuclear factor 1_cluster_27
GATA6	BATF	ARNTL	NPAS2	EGR1	RARG	SMAD4	Nuclear factor 1_cluster_44
HLTF	BATF3	ATF6	SREBF1	KLF10	NR2C2	SMAD5	Paired-related HD factors_cluster_60
MECOM	FOS	BHLHE40	SREBF2	KLF11	NR2F1	ZSCAN29	Jun-related factors_cluster_77
NFIB	FOSL1	BHLHE41	TCFL5	KLF12	NR2F6	RFX1	Jun-related factors_cluster_1
NFIC	FOSL2	CLOCK	TFE3	KLF13	RXRB	RFX2	TEF-1-related factors_cluster_2
NFIX	JDP2	CREB3	TFEB	KLF15	ESRRA	RFX3	bHLH-ZIP factors_cluster_7
HIC1	JUN	CREB3L1	USF1	KLF16	NR2C1	RFX5	CREB-related factors_cluster_18
HIC2	JUNB	CREB3L2	USF2	KLF2	NR2F2	RFX7	Runt-related factors_cluster_57
NFIA	JUND	CREB3L4	XBP1	KLF3	NR4A1	SIX1	Krüppel-related_cluster_34
THAP1	MAFK	HES1	ATF1	KLF4	NR4A2	SIX2	NR1_cluster_30
WT1	NFE2L1	HES2	ATF2	KLF5	NR6A1	TFCP2	RXR-related (NR2)_cluster_20
ZNF263	NFE2L2	HES6	ATF3	KLF6	RORA		RXR-related (NR2)_cluster_4
ZNF281	HNF4G	HIF1A	ATF7	KLF9	RORB		RXR-related (NR2)_cluster_46
NRF1	TEAD1	MAX	CREB1	MAZ	VDR		NR3_cluster_55
	TEAD2	MITF	CREB5	SP1	ESR2		SMAD factors_cluster_15
	TEAD3	MLX	CREM	SP2	PPARG		RFX-related factors_cluster_26
	TEAD4	MLXIP	GMEB2	SP3			Grainyhead-related factors_cluster_51
	ZNF684	MLXIPL		SP4			
				ZBTB14			
				ZNF148			
				ZNF740			

Supplemental Figure 5

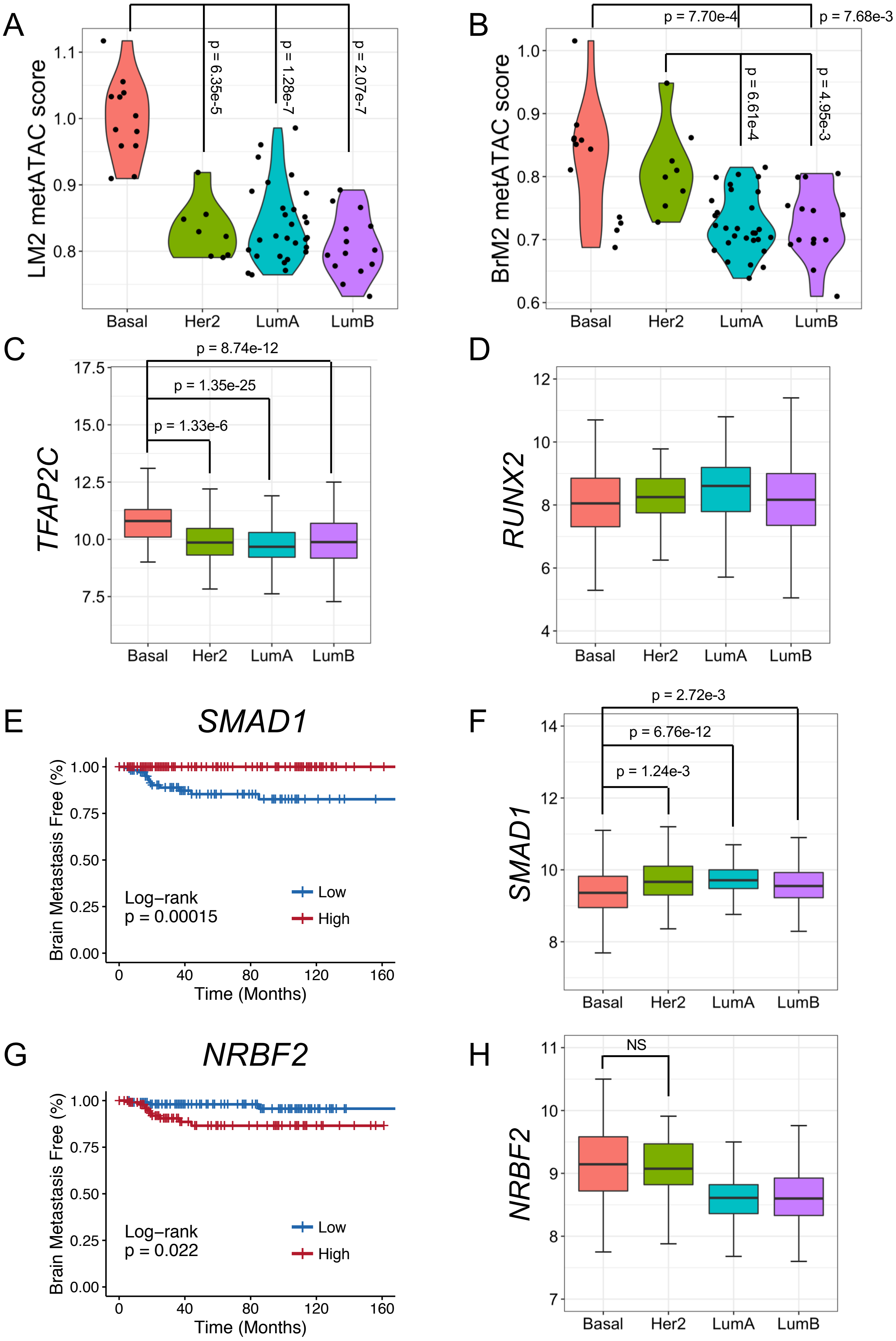

Supplemental Figure 6

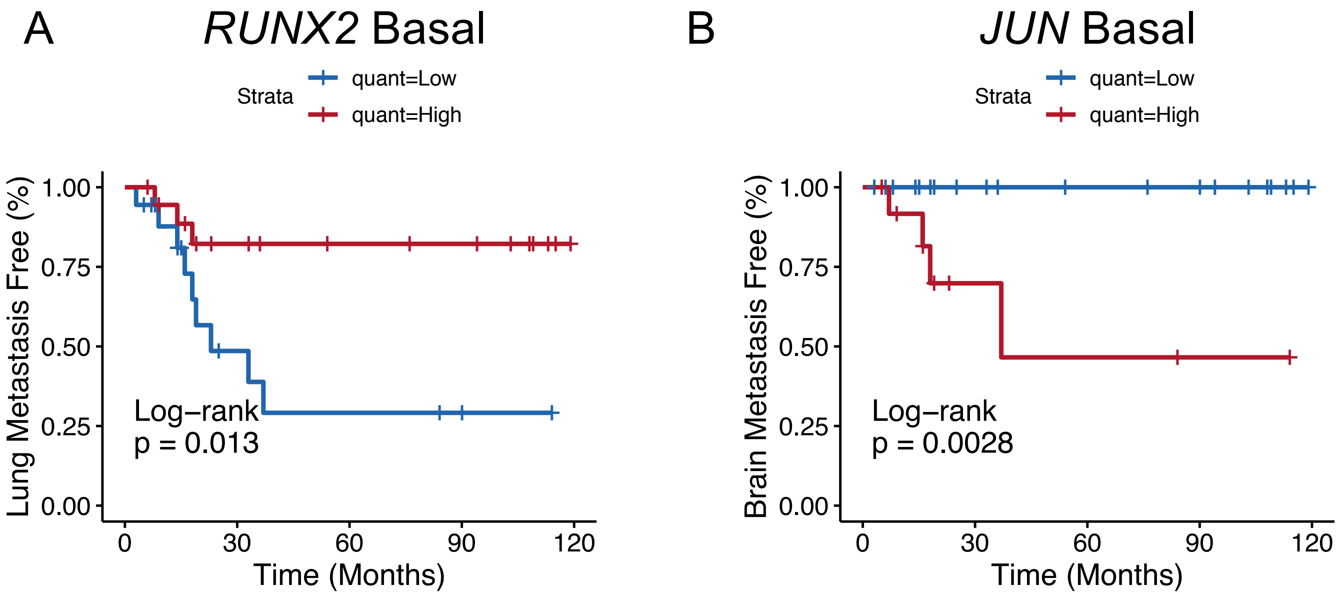

Supplemental Figure 7
